# The developmental trajectory of sleep in children with Smith-Magenis syndrome compared to typically developing peers: a 3-year follow-up study

**DOI:** 10.1093/sleepadvances/zpad034

**Published:** 2023-09-08

**Authors:** Georgie Agar, Chris Oliver, Jayne Spiller, Caroline Richards

**Affiliations:** School of Psychology, Aston University, Birmingham, UK; School of Psychology, University of Birmingham, Birmingham, UK; School of Psychology, University of Birmingham, Birmingham, UK; School of Psychology, University of Birmingham, Birmingham, UK; School of Psychology and Vision Sciences, University of Leicester, Leicester, UK; School of Psychology, University of Birmingham, Birmingham, UK; Cerebra Network for Neurodevelopmental Disorders, Birmingham, UK

**Keywords:** Smith–Magenis syndrome, actigraphy, sleep, intellectual disability, trajectory

## Abstract

**Study Objectives:**

To determine the trajectory of: (i) objective sleep parameters and (ii) caregiver-reported sleep questionnaire scores over 3 years in children with Smith-Magenis syndrome (SMS) compared to age-matched typically developing (TD) controls. We also aimed to (iii) describe individual profiles of change in sleep parameters over time.

**Methods:**

Week-long, overnight actigraphy and questionnaire data from 13 children with SMS and 13 age-matched TD children were collected at Time 1 and Time 2 (3 years later). Independent samples *t*-tests, paired samples *t*-tests, and Bayesian analyses were used to compare sleep parameters and sleep questionnaire scores between groups at each time point and compare data within groups to assess change over time.

**Results:**

Sleep parameters were consistently more disrupted in the SMS group than the TD group, with significantly reduced sleep efficiency, increased wake after sleep onset and earlier get up times at both time points. This was mirrored in the questionnaire data, with children with SMS evidencing higher scores for overall sleep disturbance, night waking, and daytime sleepiness. While TD sleep parameters demonstrated expected developmental changes over 3 years, in the SMS group sleep parameters and variability between and within children remained largely stable. However, some children with SMS showed substantial variation in sleep parameters over time. Questionnaire scores remained stable over 3 years in both groups.

**Conclusions:**

Overall, sleep disturbance appears to be a stable feature of SMS, indicative of a divergent sleep trajectory compared to TD peers. Proactive intervention approaches should be considered for poor sleep in SMS.

**Statement of Significance**

This article is the first describing the persistence of objectively defined poor sleep in individuals with Smith-Magenis syndrome (SMS), a rare genetic syndrome which affords insight into the genetic influences on sleep more broadly. In individuals with SMS, stability of all sleep parameters was noted in comparison to age-related changes to bed time and total sleep time seen in the typically developing comparison group and broader literature. Bayes factors were substantial, suggesting that poor sleep in individuals with SMS is persistent over 3 years. The overall stability of these objectively defined sleep parameters is further supported by the persistence of subjectively reported sleep disorder scores, and alludes to the potential role of the retinoic acid induced 1 (RAI1) gene in divergent sleep trajectories. Key implications for intervention are discussed.

## Introduction

Smith-Magenis syndrome (SMS) is caused by a variation or deletion to the retinoic acid induced 1 (RAI1) gene on chromosome 17p11.2. and is associated with mild to moderate intellectual disability (ID) and a well-defined behavioral phenotype of sociability, impulsivity, and elevated rates of self-injury and aggression [1–3]. Sleep disturbance is widely reported in SMS and has been delineated objectively as extended wake after sleep onset (WASO), reduced total sleep time (TST) and sleep onset latency, and earlier morning waking than age-matched typically developing (TD) peers [4]. This sleep disturbance has a demonstrable and significant impact on individuals and their caregivers [5]. Although SMS is rare, occurring in 1 in 25 000 live births [6], the population affords a window into the genetics of sleep disturbance because RAI1 is proposed to regulate the circadian locomotor output cycles kaput (“CLOCK”) gene, which in turn regulates the central circadian rhythm and several other circadian genes [7]. Therefore, understanding the profile of sleep in this syndrome has the potential to enhance understanding of sleep more broadly and inform bespoke support for people with SMS and their families.

Within pediatric populations, poor sleep is associated with poor cognitive emotional and behavioral outcomes for TD children and children with neurodevelopmental conditions such as autism and/or rare neurogenetic syndromes [8–14]. Given that SMS is associated with ID, and behavioral and emotional difficulties, (e.g. [15, 16]), there is a need to improve sleep in children in this group to mitigate such outcomes. A critical step toward determining the timing and focus of interventions is to describe the longitudinal trajectory of poor sleep in these groups, in comparison to the developmental changes in sleep well-documented in the TD population [17]. If poor sleep is persistent in SMS, more proactive, bespoke, and targeted intervention approaches may be warranted.

Despite the elevated risk of poor sleep in SMS, the developmental trajectory of sleep in this high-risk group has received limited attention in research. Using cross-sectional cohorts and clinical descriptions, both diagnosable sleep disorders and “general” sleep difficulties are demonstrated to be persistent throughout childhood and into adulthood in SMS [18], whereas these problems are generally transient in TD children [19, 20]. Cross-sectional data from informant-report diaries and sleep questionnaires suggest that as children with SMS get older they sleep less [21], wake more after sleep onset and wake earlier [22]. Although subjective methods are useful in providing a broad picture of poor sleep in these groups, they are limited by the caregiver not necessarily seeing the individual at nighttime and caregivers’ own experience of sleep deprivation [23].

In a cross-sectional study that used actigraphy as an objective sleep assessment, Trickett et al. found no relationship between TST and age in a sample of children with SMS recruited due to a caregiver reported “sleep problem” [4]. This suggests that poor sleep does not improve with age in children with SMS and supports Potocki et al.’s cross-sectional polysomnography study of 28 individuals with SMS (aged 2 to 31 years) which found no relationship between the age and TST [24]. However, a more recent cross-sectional study by Smith et al. found that WASO and early waking, as defined by actigraphy, reduced with age in individuals with SMS, indicating that some sleep parameters may improve over time [25]. Although these objective data are robust, they are limited by their cross-sectional design, and therefore, only demonstrate change (or stability) in sleep patterns without accounting for individual variation. Therefore, prospective longitudinal studies utilizing both objective and subjective sleep assessments are required to better evaluate the trajectory of poor sleep in individuals with SMS.

As sleep is a developmental process known to fluctuate through the lifespan [17, 26], it is critical to consider change or stability in sleep parameters in individuals with SMS in comparison to TD peers. There are robust data demonstrating that sleep timing alters during typical adolescence with a shift toward a later phase and later bedtime [27]. This shift is associated with a gradual reduction in overall TST as individuals get older [17]. The use of a longitudinal age-matched TD contrast group would allow researchers to quantify the severity of poor sleep in this population at high-risk for poor sleep. Furthermore, the use of a TD contrast group would enable careful evaluation of whether the divergence in sleep parameters in individuals with SMS from TD sleep parameters persists, or whether these differences remit over time.

Objective longitudinal research would also help to elucidate potential underlying mechanisms and risk markers for poor sleep in SMS, which are critical for timely and effective intervention. In SMS, the predominant explanation for poor sleep, particularly early morning waking and excessive daytime sleepiness, is the difference in the secretion pattern of melatonin [21, 24]. In comparison to TD individuals, individuals with SMS are reported to have an “inverted” melatonin secretion pattern, which peaks during the day and falls during the night, thus circadian timing and subsequent sleep parameters are *divergent* in individuals with SMS compared to TD individuals. This inverted pattern has been reported in over 90% of 27 individuals studied cross-sectionally [21, 24] and is thought to be a byproduct of dysregulation of the retinoic acid-induced (RAI1) gene, which is either deleted, or, less commonly, mutated in SMS [28]. As the genotype does not vary as the individual with SMS ages, it would be hypothesized that there would be limited variation in sleep parameters (particularly sleep onset latency and get up time) and subjectively reported sleep disorders over the lifespan. However, Smith et al. found cross-sectional improvements to WASO and reduced early waking in older children with SMS [25]. These objectively derived cross-sectional differences, together with reports of individuals with SMS who experience sleep disturbance *without* the abnormal melatonin secretion pattern (see [29]) indicate that sleep disturbance in SMS may not be caused solely by biological differences in melatonin secretion and gene regulation. Stability of objectively measured poor sleep at multiple time points would support a hypothesis of atypical and stable melatonin release as primary to poor sleep in SMS, while replication of improvements in sleep parameters would suggest that alternative mechanisms contribute to sleep outcomes and/or that atypical melatonin release is also not stable over development in individuals with SMS. These findings would have significant value more broadly in increasing understanding of the genetic pathways to sleep disturbance.

An alternative explanation for poor sleep in SMS is that rather than (or in addition to), the sleep trajectory being divergent due to a known or unknown biological mechanism it is *delayed* compared to TD peers, commensurate with the motor and cognitive delay seen in these groups (e.g. [30, 31]. If this hypothesis is correct, the same pattern of changes in sleep parameters over time as reported in the TD literature would be expected in SMS, but occurring later in children’s development, perhaps in line with mental-age or neural-age development. Galland et al.’s systematic review [17] of cross-sectional changes in 24-hour TST in TD children suggests mean sleep duration reliably reduces with age from newborns (14.6 hours) to 12-year-olds (8.9 hours). This is likely due initially to the decline in daytime napping over the first 5 years of life [32] and then later bedtimes and reduction in night sleep throughout childhood. Current cross-sectional subjective sleep data using informant-report measures similar to those synthesized by Galland et al. [17], suggest sleep parameters in SMS may mirror TD change over time [16, 21, 22] with mixed findings from actigraphy data [4, 25]. Longitudinal research is, therefore, required to consider whether sleep trajectories in SMS are delayed or divergent compared to TD peers, and in relation to developmental age, to inform intervention for poor sleep in this group.

Finally, longitudinal research is needed to explore within and between individual variability in sleep parameters over time in individuals with SMS in relation to individual characteristics. This is particularly important in rare syndromes such as SMS, to maximize the use of individual data and harness the strength of small-*N* designs in order to consider change over time [33]. Age-related changes to TST in typical development appear to be moderated by intra-person variability in environmental factors such as school timing and biological circadian phase delay [34, 35]. Therefore, poor sleep in SMS may also be influenced by other factors, such as the child’s behavior, sleep environment, and other aspects of their sleep hygiene [4, 16, 36]. The phenotypic facial features of individuals with SMS may also predispose them to sleep disordered breathing [16], which may become more or less difficult as facial characteristics mature with age [37]. Importantly, individuals with SMS show a phenotypic preference for caregiver attention [3], which may lead to multiple interactions with caregivers during the night [5, 38]. Over time, these interactions can serve to unintentionally reinforce the child’s signaling behavior at waking through the process of operant reinforcement, thus prolonging poor sleep [39]. In addition, SMS is associated with painful health conditions including constipation, reflux, and otitis media which can become chronic [18]. These conditions have been associated with poor sleep in TD individuals [40] and chronic pain more broadly is a known correlate of poor sleep in TD children [41]. As our previous work demonstrates, these nighttime interactions and painful health conditions may be partly responsible for acute poor sleep in SMS [42]. However, longitudinal research is needed to explore the relationship of child behavior, pain, and sleep hygiene to long-term poor sleep.

In summary, cross-sectional studies using subjective data collection methods indicate likely persistence of poor sleep in SMS. There is a need for a prospective longitudinal design to evaluate change in sleep parameters over time in this high-risk group, using objective assessment of sleep. In addition, TD children demonstrate age-related changes in TST and sleep consolidation over time, thus longitudinal changes in these parameters in SMS must be considered in comparison to a TD contrast group to determine whether sleep trajectories are delayed and/or divergent. In this study, we conduct a longitudinal follow-up of the cohort described by Trickett et al. [4] to delineate the developmental trajectory of poor sleep in individuals with SMS using objective measures and contrast the trajectory of change with that of TD children. The aims are:

A) To compare specific actigraphy-defined sleep parameters at Time 1 (T^1^) and Time 2 (T^2^, 3 years later) in each group to determine the trajectory of objective sleep parameters in SMS, compared to a community sample of age-matched TD controls, and determine whether they are delayed and/or divergent in individuals SMS.
B) To compare caregiver-reported sleep questionnaire scores at T^1^ and T^2^ in both groups to describe the trajectory of subjectively reported sleep disorders in SMS and compare the trajectory to that of TD peers.
C) To explore individual profiles of change in sleep parameters over time, in relation to chronological and developmental age-expected changes in sleep and reliable change in caregiver-reported sleep disorders, overactivity and impulsivity, pain, sleep hygiene, and adaptive functioning.

## Methods

### Participants

At T^1^ (2015–2016), 26 participants with SMS and 52 TD children were recruited to a longitudinal study of sleep and behavior (approved by the Science, Technology, Engineering, and Mathematics Ethical Review Committee at the University of Birmingham). All children with SMS had a confirmed genetic diagnosis according to caregiver report. The decision was taken to recruit children with SMS with a caregiver reported “sleep problem” to objectively define poor sleep in these groups (as in Trickett et al.) [4]. In addition, the recruitment of individuals with a known “sleep problem” conferred the advantage of considering possible mechanisms of poor sleep. TD children were not required to have a caregiver reported “sleep problem” to take part at T1, although by T2 some did. This decision was taken to evaluate the putative discrepancies in sleep parameters experienced by individuals with SMS with poor sleep, in comparison to what might be expected in typical development. This approach also enables consideration of how sleep parameters in SMS might differ longitudinally from a typical sleep trajectory.

The full recruitment procedure and T^1^ comparison of 20 of these children with SMS and an age-matched sample of 20 TD children, drawn from the wider cohort recruited in 2015–2016, are described in Trickett et al. [4]. At T^1^, 20 children with SMS participated in the actigraphy study, and a further three participants had informant-based assessments of sleep. Thirteen of these participants with SMS (mean age = 11.09, SD = 1.57) and 23 TD children (mean age = 9.86, SD = 2.89) took part in the follow-up study at T^2^ (2018–2019) and are the subject of this article. Of these, only 12 participants with SMS had actigraphy data available from T^1^, thus the longitudinal objective analysis has 12 participants, but longitudinal subjective analysis has 13. Supplementary Material 1 provides a summary of recruitment and attrition across both time points in both groups. There were no significant differences in T^1^ demographic or sleep characteristics between those who did and did not take part at T^2^.

A sample was selected from the TD contrast group and matched to the SMS group. Two matching approaches were trialed, in order to identify ideal matches for children with SMS based on their exact age at time of assessment (within a year) and sex. Due to the over-representation of male participants in the TD group, matching which prioritized sex resulted in all children with SMS being matched to a TD participant of the same sex, but only 7/13 being matched to a child within a year of their exact age. Matching which prioritized age resulted in all children with SMS being matched to a child within a year of their exact age, and only 5/13 *not* matched to a child of the same sex. Therefore, age-based matching was deemed the most appropriate approach in this study, especially given the comparative importance of developmental processes. The finalized matching approach is detailed in Supplementary Material 2. Table 1 describes the participant characteristics of those included in the follow-up study.

**Table 1.** Participant characteristics for each subsample

Objective analysis	SMS (*n* = 12)	TD (*n* = 12)
Mean age (SD)	11.27 (1.50)	10.83 (1.88)
Number of males	5	10
Number of females	7	2
Mode family income	£45 001–£55 000	£65 001 or more
Number taking sleep medication	8^*^	0
Mean nights of actigraphy (SD)	6.25 (1.74)	7.58 (0.67)
Subjective analysis	SMS (*n* = 13)	TD (*n* = 13)
Mean age (SD)	11.10 (1.57)	10.65 (1.91)
Number of males	6	11
Number of females	7	2
Mode family income	£45 001–£55 000	£65 001 or more
Number taking sleep medication	9^*^	0

^*^All these children were taking melatonin regularly, one child was also taking chloral hydrate, and another was taking alimemazine. One child was taking melatonin “occasionally” and is not included in this total. Only three children (in both the objective and subjective analysis) were not taking any sleep medication.

### Procedure

At T^1^, families were contacted via telephone or email to book in a “study week” where the child was asked to wear a Philips Actiwatch 2 (Philips Respironics) in their typical home environment while caregivers completed a sleep diary. All study weeks were completed during school term-time to maintain consistency and because of potential differences in term-time versus school holiday sleep patterns [43, 44]. All caregivers were advised that the actiwatch could be worn on the ankle or, preferably, the wrist, and to press the event marker at “lights out time” and “get up time.” Participants were encouraged to wear the actiwatch at all times (except for bathing and swimming, which was at caregivers’ and teachers’ discretion).

At T^2^, all participants eligible for follow-up (i.e. aged ≤15)^1^ were contacted via post with details of the longitudinal sleep study. Participants were booked in for a study week, ideally within 2 years 11 months and 3 years 1 month (1065–1125 days) of their original participation, where school term time allowed for this. The mean follow-up date was 1103 days (range: 1064–1143 days) after initial participation.

### Actigraphy assessment

Using the default settings of medium sensitivity, the actiwatch defined sleep parameters based on movement in 30-s epochs. Actigraphy data were downloaded to Philips Actiware software and cleaned using information from the caregiver sleep diary and the event marker according to the protocol outlined in Trickett et al. [4] and Agar et al. [38]. This protocol was developed to maximize accuracy of the data and remove artifact which can make actigraphy unreliable [45]. For example, sleep intervals would be excluded if the diary suggested that the watch had been removed overnight or adjusted if the diary suggested the child was sedentary rather than asleep in the early evening (see Supplementary Material 3 for the full protocol and Supplementary Material 4 for a summary of key parameters derived from actigraphy). This protocol is intended to standardize and make explicit the visual inspection process that typically occurs as part of cleaning actigraphy [46].

All data were cleaned by the first author, and 25% of participants’ data were cleaned by a research assistant to assess inter-rater reliability of the cleaning protocol. A two-way mixed-effects model inter-rater reliability analysis [47] was used to assess the absolute agreement of the two raters on each average parameter. Overall intra-class coefficients ranged from 0.921 to 0.999, thus reliability of the cleaning protocol was excellent.

### Informant-based measures

At both time points, caregivers completed the Vineland Adaptive Behavior-II Interview (VABS-II [48]) with a researcher. This was used as a measure of children’s adaptive functioning as a proxy for overall ability. In addition, caregivers completed questionnaires about their child’s behavior and characteristics.

The Modified Simonds & Parraga Sleep Questionnaire (MSPQ) [49, 50] was used as a subjective measure of children’s poor sleep with questions about the child’s sleeping environment, bedtime routine, sleep timings, history of treatment, and impact on the family. The MSPSQ has adequate internal consistency and was selected as data relating to diagnosable sleep disorders can be extracted following scoring guidelines outlined by Johnson et al. [51]. Seven subscales can be calculated: Bedtime Resistance, Sleep Onset Delay, Night Waking, Sleep Anxiety, Parasomnias, Sleep-Disordered Breathing, and Daytime Sleepiness, with test–retest reliabilities ranging from 0.83–1 [50]. In addition, a total score can be derived [51], with higher scores indicating poorer sleep. Both total and subscale scores correlate significantly with corresponding scores on the Children’s Sleep Habits Questionnaire developed by Owens et al. [52]. In addition, the Family Inventory of Sleep Habits (FISH [53]) was used as a measure of sleep hygiene, with higher scores indicating better sleep hygiene.

The Non-communicating Children’s Pain Checklist – Revised (NCCPC-R [54]) was used as a measure of pain-related behaviors. The measure is suitable for use with individuals with ID and compromised verbal communication, with excellent psychometric properties [55]. Higher scores suggest the individual is in more pain. In this study, administration was modified so that caregivers rated each behavior over a week rather than over 2 hours. This decision was taken to capture chronic but potentially intermittent pain caused by long-term health conditions, rather than bursts of acute pain. Painful health conditions are common in individuals with ID [56, 57], and this modified approach has been taken to measure “typical” pain behavior in both children and adults with ID previously [58, 59].

The Activity Questionnaire (TAQ [60]) was used to measure behavioral features associated with attention deficit hyperactivity disorder in individuals with ID. Scores are pro-rated based on an individual’s verbal ability and mobility, producing a total score and subscale scores for “overactivity,” “impulsivity” and, for verbal participants, “impulsive speech,” with higher scores indicating a greater frequency of attention-deficit hyperactivity disorder-like behaviors. The measure has robust inter-rater and test–retest reliability, and good internal consistency [60].

### Data analysis

Independent samples *t*-tests were used to compare objective and subjective sleep data between groups at each time point. Paired samples *t*-tests were used to compare data at T^1^ and T^2^ within groups to assess change over time. As some of the sleep parameters defined by actigraphy were not normally distributed, nonparametric Mann Whitney *U* and Wilcoxon rank tests were used when appropriate. Due to the number of comparisons, the alpha level was Bonferroni corrected within each family of tests.

A Coefficient of Variance (CoV) for TST, WASO, and longest period of sleep before wake was calculated for each child and group to consider variability in these parameters between individual children, and within each child’s assessment period (e.g. [4, 61]. The CoV between individual children was calculated as:group standard deviation (SD) of the variable/group mean of the variable. The CoV within each child’s assessment period was calculated as:individual SD of the variable/individual mean of the variable^2^.

To quantify the differences in objective parameters and subjectively defined sleep disorder scores between groups and over time, effect sizes were calculated (Cohen’s *d* was calculated for parametric variables and adjusted Cohen’s *d* for nonparametric variables) and Bayesian independent and paired samples *t*-tests undertaken. Bayesian statistics indicate the extent to which the data support the null hypothesis (that the groups/time points do not differ on a given variable) versus the alternative hypothesis (that the groups/time points differ), by calculating a Bayes Factor. For example, Bayesian analyses allow consideration of the change (alternative hypothesis) or stability (null hypothesis) of sleep parameters over time. This approach also improves confidence in findings drawn from small samples. Jeffreys’ [62] guidelines were used to interpret the data as per Surtees et al. [63], a study of actigraphy parameters in a sample of <20 children with autism. These guidelines suggest a Bayes Factor of 1–3 represents “anecdotal evidence” in favor of the null hypothesis, 3–10 “moderate evidence,” 10–30 “strong evidence,” 30–100 “very strong evidence,” and >100 as “extreme evidence.” Conversely, 1/3–1 represents “anecdotal evidence” in favor of the alternative hypothesis 1/10–1/3 “moderate evidence” 1/30–1/10 “strong evidence” 1/100–1/30 “very strong evidence,” and <1/100 as “extreme evidence.”

To address the final exploratory aim, individual changes to sleep parameters in the SMS group were considered in relation to chronological and developmental age-related changes in the TD sample and published normative data. Developmental age was calculated based on average age equivalent for each domain on the VABS-II at each time point. Since many children had an adaptive age equivalent below the minimum age of the TD sample (<4 years), data synthesized by Galland et al. [17] are also presented as a comparison. In addition, children were classified on each sleep parameter as either having increased or reduced sleep using visual inspection.

Questionnaire scores relating to overactivity and impulsivity, pain, caregiver reported sleep disorders, sleep hygiene, and adaptive functioning were considered in relation to individual change over time in specific sleep parameters. Given the small *n*, reliable change indices were calculated for questionnaire and interview data for each participant, using the Leeds Reliable Change Indicator [64]. Reliable change indices consider whether an individual is making reliable improvements or reductions on a given measure over time, beyond what is expected given the known test–retest reliability of the measure. The Cronbach’s alpha or intraclass coefficients were taken from the relevant manual or from published literature for each measure entered into the analyses [48, 53, 55, 65, 66].

## Results

### Group differences in actigraphy-defined sleep parameters

Table 2 shows the results of comparisons of actigraphy defined sleep parameters at T^1^ and T^2^ for children with SMS compared to age-matched TD peers.

**Table 2.** Actigraphy-defined sleep parameters, test statistics, *p*-values, effect sizes, and Bayes factors for children with SMS and age-matched TD peers at Time 1 and Time 2.

	Time 1						Time 2					
Sleep Parameter	SMS (*n* = 12)	TD (*n* = 12)	*t*/*U* score	*P-*value*	Effect size	Bayes factor	SMS (*n* = 12)	TD (*n* = 12)	*t*/*U* score	*P*-value*	Effect size	Bayes factor
Bedtime (hh:mm) Mean (SD)	20:11 (0:55)	20:46 (0:52)	1.622	.119	0.75	1.219	**19:59** **(0:57)**	**21:45** **(1:07)**	**4.178**	**<.001**	**1.77**	**.013**
Get up time (hh:mm) Mean (SD)	**05:01** **(1:14)**	**07:01** **(0:42)**	**4.924**	**<.001**	**1.94**	**.003**	**05:38** **(0:50)**	**07:04** **(0:36)**	**4.829**	**<.001**	**2.00**	**.003**
Sleep onset latency (mins) Median (IQR)	7.55 (3.05–17.73)	7.56 (4.23–17.32)	69.000	.887	0.07	3.408	14.11 (10.16–27.05)	13.24 (9.70–20.21)	64.000	.671	0.19	3.345
Wake after sleep onset (mins) Median (IQR)	70.37 (51.34–123.91)	54.08 (39.81–58.68)	35.000	.033	1.00	.417	**97.04** **(51.13**–**132.64)**	**47.66** **(32.92**–**61.47)**	**24.000**	**.005**	**1.37**	**.062**
Period of longest sleep before wake (mins) Median (IQR)	53.42 (48.90–59.22)	62.63 (55.12 –70.26)	43.000	.101	0.73	1.269	54.16 (46.41–67.31)	55.31 (51.83–68.65)	66.500	.755	0.13	3.438
Time in bed (hh:mm) Mean (SD)	**08:49** **(01:23)**	**10:15** **(0:32)**	**3.336**	**.005**	**1.37**	**.073**	09:39 (0:51)	09:19 (0:52)	−.947	.354	0.39	2.405
Sleep efficiency (%) Mean (SD)	**79.13** **(6.58)**	**86.69** **(3.53)**	**3.508**	**.003**	**1.47**	**.052**	**75.67** **(6.25)**	**85.75** **(4.48)**	**4.538**	**<.001**	**1.85**	**.006**
Total sleep time (hh:mm) Mean (SD)	**07:00** **(0:58)**	**08:53** **(0:33)**	**5.811**	**<.001**	**2.39**	**<.001**	07:16 (0:31)	07:58 (0:40)	2.859	.009	1.17	.178
CoV in total sleep time within assessment period (%) Median (IQR)	**10.70** **(8.04**–**20.64)**	**6.70** **(5.03**–**7.83)**	**24.000**	**.005**	**1.37**	**.203**	10.89 (6.27–14.33)	7.66 (4.99–8.16)	50.000	.219	0.54	1.567
Quartile-based CoV in wake after sleep onset within assessment period (%) Median (IQR)	36.65 (17.94–48.54)	30.16 (16.15–35.53)	53.000	.291	0.46	2.080	28.14 (20.50–45.30)	20.99 (17.40–29.81)	53.000	.291	0.46	1.767
Quartile-based CoV in period of longest wake within assessment period (%) Mean (SD)	21.95 (6.32)	19.37 (8.28)	−.859	.400	0.35	2.566	31.90 (12.03)	24.47 (12.40)	−1.490	.151	0.61	1.427

*p values corrected based on the Bonferonni correction, .008 for parametric and .01 for non-parametric tests. Significant differences between groups appear in bold.

At T^1^, sleep was more disrupted in the SMS group than the TD group, with children with SMS experiencing significantly poorer sleep efficiency, less time in bed and less time asleep than their TD peers. Although children in both groups went to bed at a similar time, children with SMS woke 2 hours earlier than their TD peers, with a trend toward greater WASO. However, there was no difference between the sleep onset latency (SOL) of the two groups. The Bayes factor suggests that the data are more consistent with the null hypothesis (that there is no difference between the SOL of the two groups). The CoV between children for TST and particularly for WASO was higher in the SMS group (13.8 vs. 6.3% and 103.1% vs. 34.9%, respectively). The CoV for longest period of sleep before wake was higher in the TD group (24.2% vs. 19.3%). Variability of these parameters for individual children within their own assessment period was also higher in SMS, with the CoV significantly higher for TST.

At T^2^, group differences in get up time, WASO and sleep efficiency remained significant. In addition, differences in bedtime between the groups were significant, with children with SMS going to bed 1 hour 26 minutes earlier than their TD peers. Time in bed did not differ significantly between groups (Bayes factor 2.405, “anecdotal” evidence in support of the null hypothesis), but there was a trend toward differences in TST (effect size 1.17, Bayes factor.178, “moderate” evidence in favor of the alternative hypothesis). There were no significant differences between the groups in terms of individual variability within the assessment period. Variability between children in TST (7.1% vs. 8.4%) and period of longest sleep before wake (38.6% vs. 30.4%) was also similar in both groups. Variability between children was greater for WASO in SMS (84.1% vs. 51.9%).

### Change over time in actigraphy-defined sleep parameters

To consider the developmental trajectory of poor sleep, actigraphy-defined sleep parameters at T^1^ and T^2^ in were compared for each group (Table 3). In the SMS group, children’s sleep parameters and variability between and within children remained largely stable over 3 years. Interpretation of the Bayes factors suggests “moderate” evidence in favor of the null hypothesis for bedtime, and the CoV for interassessment variability in TST and WASO, suggesting these were particularly stable. However, evidence in favor of the null hypothesis for the other variables was “anecdotal.” For the 12 children in the SMS-matched TD group, bedtime became significantly later (Bayes factor 0.001, “extreme” evidence in favor of the alternative hypothesis), while there was no change in children’s get up time. Although there was no significant change in children’s sleep efficiency, SOL or WASO, TD children did spend less time in bed as they got older and obtained significantly less TST. Levels of variability within and between children remained stable over 3 years, with “moderate” Bayes factors. Evidence in favor of the null hypothesis was also “moderate” for get up time and sleep efficiency in this group, but weaker for WASO and SOL.

**Table 3.** Change over time of actigraphy-defined sleep parameters, test statistics, *p*-values, effect sizes and Bayes factors for children with SMS and age-matched TD peers.

	SMS (*n* = 12)						TD (*n* = 12)					
Sleep parameter	Time 1	Time 2	*t*/*Z* score	*P*-value*	Effect size	Bayes factor	Time 1	Time 2	*t*/*Z* score	*P*-value*	Effect size	Bayes factor
Bedtime (hh:mm) Mean (SD)	20:11 (0:55)	19:59 (0:57)	.858	.409	0.25	3.314	**20:46** **(0:52)**	**21:45** **(1:07)**	**−6.781**	**<.001**	**0.88**	**.001**
Get up time (hh:mm) Mean (SD)	05:01 (1:14)	05:38 (0:50)	–2.088	.061	0.49	.812	07:01 (0:42)	07:04 (0:36)	−.529	.607	0.07	4.081
Sleep onset latency (mins) Median (IQR)	7.55 (3.05–17.73)	14.11 (10.16–27.05)	–2.315	.021	1.07	.391	7.56 (4.23–17.32)	13.24 (9.70–20.21)	−1.255	.209	0.53	2.668
Wake after sleep onset (mins) Median (IQR)	70.37 (51.34–123.91)	97.04 (51.13–132.64)	–1.098	.272	0.46	2.600	54.08 (39.81–58.68)	47.66 (32.92–61.47)	−1.804	.071	0.79	.989
Period of longest sleep before wake (mins) Median (IQR)	53.42 (48.90–59.22)	54.16 (46.41–67.31)	–.235	.814	0.10	3.427	62.63 (55.12–70.26)	55.31 (51.83–68.65)	−1.569	.117	0.67	2.539
Time in bed (hh:mm) Mean (SD)	08:49 (01:23)	09:39 (0:51)	–2.290	.043	0.73	.607	**10:15** **(0:32)**	**09:19** **(0:52)**	**5.115**	**<.001**	**1.30**	**.009**
Sleep efficiency (%) Mean (SD)	79.13 (6.58)	75.67 (6.25)	1.843	.092	0.54	1.137	86.69 (3.53)	85.75 (4.48)	1.549	.583	0.67	3.355
Total sleep time (hh:mm) Mean (SD)	07:00 (0:58)	07:16 (0:31)	–1.005	.336	0.34	2.936	**08:53** **(0:33)**	**07:58** **(0:40)**	**4.485**	**.001**	**1.50**	**.022**
Coefficient of variance in total sleep time within assessment period (%) Median (IQR)	10.70 (8.04–20.64)	10.89 (6.27–14.33)	–.863	.388	0.36	3.945	6.70 (5.03–7.83)	7.66 (4.99–8.16)	−.549	.583	0.23	3.602
Quartile-based coefficient of variance in wake after sleep onset within assessment period (%) Median (IQR)	36.65 (17.94–48.54)	28.14 (20.50–45.30)	–.863	.388	0.36	3.283	30.16 (16.15–35.53)	20.99 (17.40–29.81)	−.628	.530	0.26	3.412
Quartile-based coefficient of variance in period of longest wake within assessment period (%) Mean (SD)	21.95 (6.32)	31.90 (12.03)	−2.879	.015	1.04	.250	19.37 (8.28)	24.47 (12.40)	−1.628	.132	0.48	1.502

*p values corrected based on the Bonferonni correction, .008 for parametric and .01 for non-parametric tests. Significant differences appear in bold.

### Group differences in subjectively defined sleep disorders

Table 4 shows the group comparisons for subjectively defined sleep disorders and sleep hygiene scores. Caregivers of children with SMS reported higher overall MSPSQ scores and higher subscale scores for night waking and daytime sleepiness than caregivers of age-matched TD children at both time points, despite comparable sleep hygiene scores, with “extreme” evidence for the alternative hypothesis. However, it should be noted that the mean MSPSQ score in both groups at T^1^ was above the cutoff of 56 for “poor sleepers” as suggested by Johnson et al. [51]. At T^1^, children with SMS were also reported to have higher scores on the parasomnias subscale, but this difference was not significant at T^2^. No significant differences were found between groups for bedtime resistance, sleep onset delay, sleep anxiety and sleep disordered breathing at either time point. Interpretation of the Bayes factors suggests evidence for the null hypothesis was “anecdotal” in all cases, except for bedtime resistance and sleep anxiety at T^1^ and sleep onset delay at T^2^ where evidence for the null hypothesis was “moderate” (Table 4).

**Table 4.** Subjectively defined sleep disorders and sleep hygiene scores, test statistics, *p*-values, effect sizes, and Bayes factors for children with SMS and age-matched TD peers at Time 1 and Time 2.

	Time 1						Time 2					
Sleep Questionnaire Score	SMS (*n* = 13)	TD (*n* = 13)	*t*/*U* score	*P-*value*	Effect size	Bayes factor	SMS (*n* = 13)	TD (*n* = 13)	t/*U* score	*P*-value*	Effect size	Bayes factor
Bedtime resistance Mean (SD)	12.38 (4.29)	12.08 (2.99)	−.212	.834	0.08	3.527	12.31 (4.99)	10.31 (3.75)	−1.155	.259	0.45	2.071
Sleep onset delay Mean (SD)	2.08 (.95)	1.77 (1.17)	−.7 36	.469	0.29	2.864	2.08 (.95)	1.85 (1.14)	−.559	.582	0.22	3.152
Sleep anxiety Mean (SD)	10.69 (3.22)	11.23 (1.79)	.527	.603	0.21	3.199	11.08 (4.11)	8.69 (2.84)	−1.720	.098	0.68	1.094
Night waking Median (IQR)	**8.00** **(6.5–8)**	**4.00** **(2-4.5)**	**3.000**	**<.001**	**2.86**	**<.001**	**7.00** **(7-8)**	**4.00** **(2-4.5)**	**0.000**	**<.001**	**3.23**	**<.001**
Parasomnias Mean (SD)	**24.92** **(7.44)**	**17.08** **(5.48)**	−**3.060**	**.005**	**1.20**	**.118**	22.15 (6.28)	16.15 (4.65)	−2.767	.011	1.09	.206
Sleep disordered breathing Mean (SD)	10.00 (3.27)	6.92 (2.36)	−2.753	.011	1.08	.211	8.85 (2.76)	6.69 (2.39)	−2.124	.044	0.84	.614
Daytime sleepiness Mean (SD)	**7.08** **(1.67)**	**2.92** **(1.75)**	−**6.208**	**<.001**	**2.43**	**<.001**	**7.00** **(1.63)**	**3.38** **(1.66)**	−**5.598**	**<.001**	**2.20**	**.001**
Modified Simonds and Parraga Sleep Questionnaire Total Mean (SD)	**88.54** **(11.02)**	**59.61** **(15.74)**	−**5.427**	**<.001**	**2.13**	**.001**	**83.62** **(9.06)**	**54.69** **(13.53)**	−**6.404**	**<.001**	**2.51**	**<.001**
Family Inventory of Sleep Habits Total Mean (SD)	46.46 (5.29)	50.46 (3.95)	2.186	.039	0.86	.557	45.23 (4.42)	47.77 (5.05)	1.364	.185	0.54	1.679

*p values corrected based on the Bonferonni correction, .006 for parametric and .05 for non-parametric tests. Significant differences appear in bold.

### Change over time in subjectively defined sleep disorders

The results of the analysis of change over time in subjectively defined sleep disorders and sleep hygiene scores are shown in Table 5. In the SMS group, caregiver reported sleep hygiene and sleep disorders were largely stable over time, with no significant differences between T1 and T2 scores. Evidence was moderate for the null hypothesis for all scores, except for the MSPSQ total score and parasomnia subscale score (Table 5). Age-matched TD peers showed a similar stability of subscale scores. Interpretation of the Bayes factor suggests “moderate” evidence in favor of the null hypothesis for sleep onset delay, sleep-disordered breathing, daytime sleepiness, and night waking but weaker evidence for the stability of the other subscales.

**Table 5.** Change over time in subjectively defined sleep disorders and sleep hygiene scores, test statistics, *p*-values, effect sizes, and Bayes factors for children with SMS and age-matched TD peers.

	SMS (*n* = 13)						TD (*n* = 13)					
Sleep Questionnaire Score	Time 1	Time 2	*t*/*Z*-score	*P*-value*	Effect size	Bayes factor	Time 1	Time 2	*t*/*Z*-score	*P*-value*	Effect size	Bayes factor
Bedtime resistance Mean (SD)	12.38 (4.29)	12.31 (4.99)	.068	.947	0.02	4.816	12.08 (2.99)	10.31 (3.75)	1.735	.108	0.52	1.340
Sleep onset delay Mean (SD)	2.08 (.95)	2.08 (.95)	<.001	1.000	0.00	4.827	1.77 (1.17)	1.85 (1.14)	−1.000	.337	0.07	3.047
Sleep anxiety Mean (SD)	10.69 (3.22)	11.08 (4.11)	−.534	.603	0.11	4.218	11.23 (1.79)	8.69 (2.84)	2.649	.021	1.07	.349
Night waking Median (IQR)	8.00 (6.5-8)	7.00 (7-8)	−.250	.803	0.10	4.686	4.00 (2-4.5)	4.00 (2-4.5)	-.574	.566	0.23	4.581
Parasomnias Mean (SD)	24.92 (7.44)	22.15 (6.28)	1.459	.170	0.40	1.893	17.08 (5.48)	16.15 (4.65)	1.209	.250	0.18	2.495
Sleep disordered breathing Mean (SD)	10.00 (3.27)	8.85 (2.76)	1.015	.330	0.38	3.008	6.92 (2.36)	6.69 (2.39)	.415	.686	0.10	4.447
Daytime sleepiness Mean (SD)	7.08 (1.67)	7.00 (1.63)	.154	.880	0.05	4.772	2.92 (1.75)	3.38 (1.66)	−.683	.508	0.27	3.876
Modified Simonds and Parraga sleep questionnaire Total Mean (SD)	88.54 (11.02)	83.62 (9.06)	1.946	.076	0.49	1.005	59.61 (15.74)	54.69 (13.53)	2.086	.059	0.34	.822
Family Inventory of Sleep Habits Total Mean (SD)	46.46 (5.29)	45.23 (4.42)	.925	.373	0.25	3.250	50.46 (3.95)	47.77 (5.05)	2.604	.023	0.59	.374

*p values corrected based on the Bonferonni correction, .006 for parametric and .05 for non-parametric tests.

### Individual change over time

**Figure 1.**
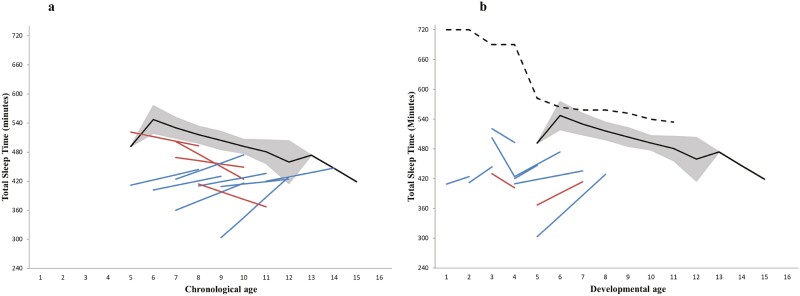
Changes in TST for children with SMS over 3 years. Each line depicts a child with SMS’s TST at T^1^ and T^2^. The black line indicates the mean TST of children in the TD contrast group at each age, with 95% confidence intervals plotted in gray. In (A), sleep trajectory is considered in relation to individuals’ chronological age. The red lines represent children with SMS who showed a decrease in mean TST over 3 years, and the blue lines those who showed an increase in mean TST. In (B), sleep trajectory is considered in relation to individuals’ nearest developmental age, according to the VABS-II. The blue lines represent children whose developmental age increased over 3 years, and the red lines those who evidence a regression in developmental age over 3 years. Two participants are not depicted—one whose VABS-II data were missing at T^1^ and one who evidenced no change in developmental age over 3 years. The dashed black line represents data reported at each age by Galland et al. (2012), reflecting observed TST for children younger than four.

Given individual variation in objective sleep parameters within the SMS group identified in the CoV analysis, each child’s data were compared at T^1^ and T^2^ in relation to their chronological *and* developmental ages, and the mean TST at each age from the TD contrast group and published normative data, to further consider whether the trajectory of TST is delayed or divergent in SMS. Figure 1 presents the TST of each individual child with SMS at T^1^ and T^2^ in relation to their chronological age Figure 1, A and their developmental age Figure 1, B. As indicated by red and blue lines in Figure 1, A, although mean TST did not change over 3 years for the SMS group (Table 3), it did decrease for some individual children (*n* = 4) and increase for others (*n* = 8). Generally, TST did not fall within the TD confidence intervals and in all cases, children with SMS were receiving less sleep than would be expected for their developmental age Figure 1, B.

To consider age-related changes in sleep consolidation, each child’s individual WASO was compared at T^1^ and T^2^ in relation to changes to their chronological and developmental age.

**Figure 2.**
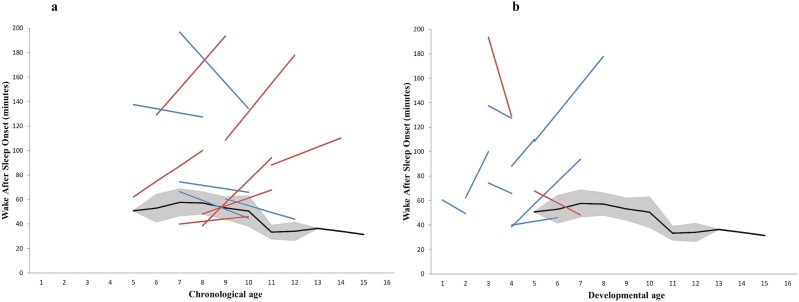
Changes in WASO for children with SMS over 3 years. Each line depicts a child with SMS’s WASO at T^1^ and T^2^. The black line indicates the mean WASO of children in the TD contrast group at each age, with 95% confidence intervals plotted in gray. In (A) changes to WASO are considered in relation to individuals’ chronological age. The red lines represent children with SMS who showed an increase in mean WASO over 3 years, and the blue lines those who showed a decrease in mean WASO. In (B) WASO trajectory is considered in relation to individuals’ nearest developmental age, according to the VABS-II. The blue lines represent children whose developmental age increased over 4 years, and the red lines those who evidence a regression in developmental age over 3 years. Two participants are not depicted—one whose VABS-II data were missing at T^1^ and one who evidenced no change in developmental age over 3 years.

Figure 2 presents the mean WASO of each individual child with SMS at T^1^ and T^2^ in relation to their chronological age Figure 2, A and their developmental age Figure 2, B. As indicated by red and blue lines in Figure 2, A, although mean WASO did not change over 3 years for the SMS group (Table 3), it did decrease for some individual children (*n* = 5) and increase for others (*n* = 7). Some individuals’ mean WASO fell within the TD confidence intervals, but generally children with SMS were awake for longer than their chronologically age-matched TD peers Figure 2, A and for longer than expected given their developmental age Figure 2, B.

In summary, although group means revealed stability of objective sleep parameters and subjectively defined sleep disorders, visual inspection of the data suggests some individual children demonstrated a change in objectively defined TST and WASO.

To address the final aim of the study, change in mean sleep parameters for each child with SMS is summarized in Table 6. Increased TST was generally accompanied by later get up times and earlier bedtimes, rather than reduced WASO. Decreased TST was accompanied by increased SOL and later bedtimes. Reliable change statistics were calculated for each individual participant on factors where individual variability, above and beyond chronological and developmental age, might be associated with a change in sleep parameters. As demonstrated in Table 6, there were no discernible patterns of change observed via visual inspection associated with experiencing an increase or decrease in any objectively defined sleep parameters.

**Table 6.** Reliable change in child characteristics from T^1^ to T^2^ in relation to increases or decreases in mean TST, mean WASO, mean bed time, mean get up time, and mean sleep onset latency in the SMS group.

Participant	Exact age at T^2^	Mean change in total sleep time	Mean change in wake after sleep onset	Mean change in bedtime	Mean change in get up time	Mean change in SOL	Melatonin T1	Melatonin T2	TAQ	NCPCC-R	MSPSQ	FISH	VABS Commu­nication	VABS daily living skills	VABS socialization	VABS motor
PPT1	12.21	**+02:05:26**	*+69.29*	**−01:28:18**	**+01:44:56**	*+2*	~	~	O	−	O	−	O	O	O	+
PPT2	10.48	**+00:56:18**	**−21.73**	**−00:51:27**	**+00:27:11**	*+1.3*	N	N	−	−	O	O	M	M	M	M
PPT3	10.38	**+00:50:37**	*+6.29*	**−00:00:53**	**+01:03:04**	*+3.48*	Y	Y	+	O	O	O	O	+	O	O
PPT4	10.99	**+00:32:19**	*+37.8*	**−01:06:50**	**+00:21:27**	*+11.13*	Y	Y	O	+	O	O	O	O	O	O
PPT5	9.94	**+00:28:20**	*+64.5*	**−00:10:20**	**+01:22:50**	**−3.48**	N	Y	−	+	O	O	O	O	−	O
PPT6	14.99	**+00:27:06**	*+21.9*	**−00:36:26**	**+00:20:32**	**−1.9**	Y	Y	+	+	O	O	O	O	O	O
PPT7	11.84	**+00:25:26**	*+55.5*	**−00:09:17**	**+01:37:47**	*+22.5*	Y	Y	+	+	O	O	O	−	O	O
PPT8	12.07	**+00:14:33**	**−16.5**	*+00:41:00*	**+00:49:40**	*+11.11*	Y	Y	−	−	+	O	O	O	O	O
PPT9	10.87	*−00:20:37*	**−62.43**	**-00:50:49**	−*01:55:19*	*+4.87*	Y	Y	O	+	O	O	−	O	−	O
PPT10	8.99	*−00:28:25*	**−10.16**	*+00:35:20*	**+00:20:10**	*+11.42*	N	N	O	O	O	−	−	+	−	O
PPT11	11.76	*−00:46:37*	*+19.44*	*+00:44:45*	**+01:32:46**	*+3.88*	Y	Y	+	O	O	O	−	O	−	−
PPT12	10.67	*−01:18:05*	**−8.38**	*+00:51:42*	*−00:19:48*	**−0.43**	N	N	−	O	O	O	+	O	O	O

Variables where an “increase” or “decrease” would be associated with greater TST are indicated in bold, and variables where an “increase” or “decrease” would be associated with reduced TST are indicated in italics. Similarly, “earlier” bedtimes and “later” get up times theoretically increase TST, while “later” bedtimes and “earlier” get up times reduce TST. It is acknowledged that there are circumstances where these changes could otherwise be problematic (i.e. oversleeping or going to bed before dinner), but they are presented in this way to provide a simple overview of the data.

Abbreviations: Y: yes; N: no; ~: occasional melatonin use; O: no reliable change; −: reliable decline; +: reliable improvement; M: missing data.

## Discussion

This article demonstrates the 3-year trajectory of objectively defined sleep parameters and caregiver reported sleep disorders in SMS, a syndrome at ultra-high risk for poor sleep, in comparison to TD age-matched peers. This was the first longitudinal study to use actigraphy, a validated objective measure of sleep, to compare sleep parameters in children with SMS to TD chronologically age-matched peers. This strengthens the validity of findings and demonstrates the utility of this assessment approach. The use of in-depth analysis to consider group and individual differences in sleep profiles maximizes the data derived from a modest sample of participants with an exceptionally rare syndrome. This represents a robust and rigorous approach to phenotype sleep and changes in sleep over time in studies with clinical samples that are often rare and prone to attrition. The results of this study extend previous objective and subjective research suggesting poor sleep is stable in individuals with SMS, and consistently worse than TD peers, by demonstrating no significant change in objective atypical sleep parameters and subjectively defined sleep disorders over 3 years. This stability contrasts with typical age-expected changes to TST, sleep consolidation, and sleep onset and offset times. In particular, children with SMS showed persistent reductions in TST and sleep efficiency, earlier bed and get up times and greater WASO than TD peers, at both T^1^ and T^2^.

The results of the study demonstrate stability of sleep parameters in children with SMS who were recruited due to a “sleep problem” 3 years after initial assessment. In contrast to reductions in TST with age reported in the literature and demonstrated by TD peers, on average children with SMS had stable bedtimes and get up times over 3 years and did not show a longitudinal reduction in TST. WASO remained stable in SMS and TD samples but was significantly higher in the SMS group than the TD group at T^2^, suggesting that sleep does not become more consolidated over time. In the SMS group, average get up times were 2 hours earlier than TD peers at T^1^ and 90 minutes earlier at T^2^, demonstrating a statistically significant divergent sleep trajectory in those with SMS recruited for poor sleep. The stability in this subsample contrasts with cross-sectional reports of individuals with SMS waking more and earlier [22] or more and later [25] as they get older but supports cross-sectional polysomnography and actigraphy data demonstrating no relationship between age and TST [4, 24]. The stability of poor sleep in SMS is further supported by the persistence of individuals’ subjectively defined sleep disorder scores, with moderate evidence for the null hypothesis on most subscales of the MSPSQ including sleep disordered breathing. Importantly, these differences were noted despite equivalent sleep hygiene scores with the TD group at both time points.

Taken together, data from these objective and subjective sleep assessments suggest poor sleep does not naturally remit over time in children with SMS and thus proactive intervention approaches should be considered for poor sleep in this group. The stability of the objective and subjective sleep data in individuals with SMS itself is not typical, as highlighted by reductions in time in bed and TST and later bedtimes in the matched TD contrast group (and wider TD literature, see [17]). This suggests a divergent sleep trajectory in SMS compared to TD peers, rather than a delay in the emergence of a typical sleep profile. This hypothesis is further supported by the notable disparity between the TST of individuals with SMS and those observed at equivalent developmental ages by Galland et al. [17], suggesting that there is not a “delay” in the acquisition of sleep consolidation in this group. However, it should be noted that, while not “typical,” the lack of age-expected reduction in TST in SMS may be a positive finding, given that children were already sleeping less than TD peers at T^1^, with SMS TST below the 95% confidence intervals of the TD mean at each chronological age. Therefore, further reduction in TST over time would not be beneficial for children with SMS.

The stability of the early get up time in the SMS group across 3 years, coupled with relatively short and stable SOL provides further support for a *divergent* sleep trajectory in SMS; a stable but inverted melatonin secretion pattern which causes individuals to feel sleepy in the day and more awake after 03:00 am [21, 24]. This is likely due to the downstream effects of RAI1 dysregulation to the CLOCK and other circadian genes [7]. Furthermore, stability of the average bedtime in the SMS group (with moderate Bayes factor) suggests children now need to go to bed earlier than is typical, arguably because they feel tired much earlier than TD peers. In addition, it may be that caregivers are still keen to implement earlier bedtimes for children with SMS than what might be considered “typical,” given the profile of early waking and the significant difficulties associated with caring for people with SMS and keeping them safe overnight [5].

However, it should be noted that although data based on group means revealed stability of objective sleep parameters and subjectively defined sleep disorders, visual inspection of the data suggests some individual children demonstrated a change in objectively defined TST and WASO which is likely to be significant to those children and their caregivers. Interestingly, a substantial proportion of children with SMS demonstrated an increase in TST at T2 with some experiencing later get up times, reduced WASO and SOL and earlier bedtimes. This suggests that while the group means for these parameters are stable (and indeed for some individual children become markedly poorer over time), there is individual variability in parameters and the possibility of clinically meaningful improvements over time.

In addition, the data highlighted substantial intraindividual and interindividual variation in sleep parameters within individuals with the same genetic syndrome. Variability between children in WASO for example was much greater in the SMS group than the TD group at both time points, suggesting waking is more problematic for some children than others. To consider which factors might be associated with these individual differences, reliable change statistics for several measures relating to child melatonin use, behavioral and adaptive characteristics and possible indicators of pain in relation to changes in sleep parameters were conducted. However, no obvious pattern of reliable improvements or deteriorations associated with changes in sleep parameters could be identified from the exploratory reliable change indices, and the relatively small sample size prevented other inferential statistical approaches. Of particular note is the suggestion that exogenous melatonin use is not accompanied by sustained improvement or worsening in sleep parameters in either group. This is particularly surprising given the number of children in this study taking exogenous melatonin to improve sleep, and the reported disruption to the endogenous melatonin cycle in individuals with SMS [21]. A further limitation of the study is that without the use of polysomnography, it is not possible to rule out the influence of periodic limb movements and sleep-related breathing difficulties on sleep parameters in SMS [67]. However, polysomnography may not be accessible for many individuals with SMS due to anxiety around sleeping in an unfamiliar environment, or tolerating the equipment required [68]. It is, therefore, not yet clear why some individuals’ TST or WASO might increase over 3 years while others’ decrease, but future research should aim to better characterize these changes utilizing more objective measures of children’s overnight breathing, movement, behavioral and pain-related characteristics, and circadian rhythm analyses, in addition to actigraphy. Understanding this variability is likely to be of great importance to families who cite sleep as a key informational need and is crucial for improving syndrome-related interventions [5].

It is acknowledged that while the longitudinal design enabled researchers to consider the trajectory of sleep parameters and subjectively defined sleep disorders in a high-risk rare syndrome, this approach did limit the sample to those who were able and motivated to take part in a comprehensive at-home sleep assessment, twice. Several families were not able to commit to such an extensive study having already participated 3 years previously, and one participant with SMS whose family did want to participate again struggled to tolerate the actiwatch 3 years later. Therefore, the data presented here may reflect the sleep profiles of only the most motivated families, or the children with the least sensory difficulties or “challenging” behavior at bedtime. However, given the range of reliable gains and declines on several measures of child behavior and adaptive ability, alongside individual changes to sleep parameters, this seems somewhat unlikely. Retention of TD children across both timepoints was also a challenge, with only 23 complete actigraphy assessments at both T^1^ and T^2^ and 17 of these from male participants. As age was the priority variable for matching, this, therefore, meant that a disproportionate number of males were included in the control sample and five females with SMS had to be matched to males. This imbalance in participants’ sex may have influenced the extent of the differences between the SMS and TD groups. Further research should explore the potential role of sex differences in sleep parameters in SMS. Furthermore, the design of the study, a longitudinal analysis of sleep in *children* with SMS, meant that it was not possible to consider individuals under the age of 4 or over 15 at either time point. Therefore, the data do not reflect poor sleep at every age of childhood, and some older children recruited at T^1^ could not be included at T^2^, limiting the sample size and application of the findings to change over time in children aged 4 to 15 only. Further research should investigate the sleep profiles of both younger and older individuals with SMS to delineate a sleep trajectory across the full lifespan.

The size of the sample was modest, due to the rarity of the syndrome and some attrition over the 3-year data collection period, which did limit the analysis approaches that could be undertaken in this study (e.g. planned regression analyses could not be conducted to predict changes to sleep parameters at T2). The remaining frequentist analyses should be interpreted with appropriate caution until future studies have replicated these findings in larger samples. However, a key strength of the present study is the application of Bayesian approaches, which allow quantification of the support for the null versus alternative hypotheses even in small samples. Bayesian statistics have recently been applied to sleep research in individuals with autism [63] and may be beneficial for future studies of other neurodevelopmental conditions where recruitment and retention of large sample sizes may be particularly difficult.

In conclusion, this study is the first to compare the longitudinal sleep trajectory of children with SMS to TD age-matched controls using objective actigraphy data. Comparison of sleep parameters revealed poorer sleep at both time points in the SMS group, and stability of parameters over 3 years. This stability, in contrast to TD age-related changes to TST, sleep consolidation, bedtime, and get up time, suggests a divergent sleep trajectory in SMS. This may be driven by a biological change, such as an altered circadian rhythm [21, 24]. The objective data are further supported by the stability of caregiver reported sleep disorders in SMS, which were elevated compared to TD peers. However, for some individual children substantial improvements to TST, WASO, and SOL are noted, alongside changes to bedtimes and get up times. These changes did not seem to be associated with specific child behaviors or adaptive functioning. Taken together, these findings suggest that poor sleep is not transient in individuals with SMS, thus proactive intervention is warranted.

## Supplementary Material

zpad034_suppl_Supplementary_Materials

## Data Availability

The data in this article cannot be publicly shared due to ethical reasons.

## Footnotes

1 Note that ethical approvals only allowed children under the age of 16 to participate at both T^1^ and T^2^. Therefore, a cohort of children who had been eligible to take part at T^1^ were no longer eligible at T^2^ and were, therefore, not approached for the follow-up data collection.

2 For nonparametric variables a Quartile-based Coefficient of Variance (QCV) was calculated, using the interquartile range and median in place of the SD and mean, respectively.

## References

[1] Arron K , Oliver C, Moss J, Berg K, Burbidge C. The prevalence and phenomenology of self-injurious and aggressive behaviour in genetic syndromes. J Intellect Disabil Res. 2011;55(2):109–120. doi:10.1111/j.1365-2788.2010.01337.x. 20977515

[2] Oliver C , Berg K, Moss J, Arron K, Burbidge C. Delineation of behavioral phenotypes in genetic syndromes: characteristics of autism spectrum disorder, affect and hyperactivity. J Autism Dev Disord. 2011;41(8):1019–1032. doi:10.1007/s10803-010-1125-5. 21080217

[3] Wilde L , Silva D, Oliver C. The nature of social preference and interactions in Smith-Magenis syndrome. Res Dev Disabil. 2013;34(12):4355–4365. doi:10.1016/j.ridd.2013.09.014. 24120292

[4] Trickett J , Oliver C, Heald M, et al. Sleep in children with Smith-Magenis syndrome: a case-control actigraphy study. Sleep. 2020;43(4):zsz260. doi:10.1093/sleep/zsz260. 31630201

[5] Agar G , Bissell S, Wilde L, et al. Caregivers’ experience of sleep management in Smith–Magenis syndrome: a mixed-methods study. Orphanet J Rare Dis. 2022;17(1):35. doi:10.1186/s13023-021-02159-8. 35120534 PMC8815225

[6] De Leersnyder H. Chapter 34 - Smith–Magenis syndrome. In: Dulac O, Lassonde M, Sarnat HB, eds. Handbook of Clinical Neurology. Vol 111. Pediatric Neurology Part I. Elsevier; 2013:295–296. doi:10.1016/B978-0-444-52891-9.00034-8. 23622179

[7] Williams SR , Zies D, Mullegama SV, Grotewiel MS, Elsea SH. Smith-Magenis syndrome results in disruption of CLOCK gene transcription and reveals an integral role for RAI1 in the maintenance of circadian rhythmicity. Am J Hum Genet. 2012;90(6):941–949. doi:10.1016/j.ajhg.2012.04.013. 22578325 PMC3370274

[8] Didden R , Korzilius H, van Aperlo B, van Overloop C, de Vries M. Sleep problems and daytime problem behaviours in children with intellectual disability. J Intellect Disabil Res. 2002;46(Pt 7):537–547. doi:10.1046/j.1365-2788.2002.00404.x. 12354310

[9] Edgin JO , Tooley U, Demara B, Nyhuis C, Anand P, Spanò G. Sleep disturbance and expressive language development in preschool-age children with down syndrome. Child Dev. 2015;86(6):1984–1998. doi:10.1111/cdev.12443. 26435268 PMC4626407

[10] Fallone G , Acebo C, Seifer R, Carskadon MA. Experimental restriction of sleep opportunity in children: effects on teacher ratings. Sleep. 2005;28(12):1561–1567. doi:10.1093/sleep/28.12.1561. 16408416

[11] Gruber R , Laviolette R, Deluca P, Monson E, Cornish K, Carrier J. Short sleep duration is associated with poor performance on IQ measures in healthy school-age children. Sleep Med. 2010;11(3):289–294. doi:10.1016/j.sleep.2009.09.007. 20156702

[12] Li S , Arguelles L, Jiang F, et al. Sleep, school performance, and a school-based intervention among school-aged children: a sleep series study in China. PLoS One. 2013;8(7):e67928. doi:10.1371/journal.pone.0067928. 23874468 PMC3707878

[13] Paavonen EJ , Porkka-Heiskanen T, Lahikainen AR. Sleep quality, duration and behavioral symptoms among 5–6-year-old children. Eur Child Adolesc Psychiatry. 2009;18(12):747–754. doi:10.1007/s00787-009-0033-8. 19466475

[14] Touchette E , Petit D, Séguin JR, Boivin M, Tremblay RE, Montplaisir JY. Associations between sleep duration patterns and behavioral/cognitive functioning at school entry. Sleep. 2007;30(9):1213–1219. doi:10.1093/sleep/30.9.1213. 17910393 PMC1978413

[15] Oliver C , Adams D, Allen D, et al. Causal models of clinically significant behaviors in angelman, cornelia de lange, prader-willi and smith-magenis syndromes. In: Hastings R, Rojahn J, eds. International Review of Research in Developmental Disabilities: Challenging Behavior, Vol 44. International Review of Research in Developmental Disabilities. Elsevier Academic Press; 2013:167–211. doi:10.1016/B978-0-12-401662-0.00006-3.

[16] Trickett J , Heald M, Oliver C, Richards C. A cross-syndrome cohort comparison of sleep disturbance in children with Smith-Magenis syndrome, Angelman syndrome, autism spectrum disorder and tuberous sclerosis complex. J Neurodev Disord. 2018;10(1):9. doi:10.1186/s11689-018-9226-0. 29490614 PMC5831859

[17] Galland BC , Taylor BJ, Elder DE, Herbison P. Normal sleep patterns in infants and children: a systematic review of observational studies. Sleep Med Rev. 2012;16(3):213–222. doi:10.1016/j.smrv.2011.06.001. 21784676

[18] Gropman AL , Duncan WC, Smith ACM. Neurologic and developmental features of the smith-magenis syndrome (del 17p11.2). Pediatr Neurol. 2006;34(5):337–350. doi:10.1016/j.pediatrneurol.2005.08.018. 16647992

[19] Gregory AM , O’connor TG. Sleep problems in childhood: a longitudinal study of developmental change and association with behavioral problems. J Am Acad Child Adolesc Psychiatry. 2002;41(8):964–971. doi:10.1097/00004583-200208000-00015. 12162632

[20] Quach J , Hiscock H, Canterford L, Wake M. Outcomes of child sleep problems over the school-transition period: Australian population longitudinal study. Pediatrics. 2009;123(5):1287–1292. doi:10.1542/peds.2008-1860. 19403493

[21] De Leersnyder H , de Blois MC, Vekemans M, et al. Beta1-adrenergic antagonists improve sleep and behavioural disturbances in a circadian disorder, Smith-Magenis syndrome. J Med Genet. 2001;38(9):586–590. doi:10.1136/jmg.38.9.586. 11546826 PMC1734944

[22] Smith ACM , Dykens E, Greenberg F. Sleep disturbance in Smith-Magenis syndrome (del 17 p11.2). Am J Med Genet. 1998;81(2):186–191. doi:10.1002/(SICI)1096-8628(19980328)81:2<186::AID-AJMG11>3.0.CO;2-D. 9613860

[23] Short MA , Gradisar M, Lack LC, Wright HR, Chatburn A. Estimating adolescent sleep patterns: parent reports versus adolescent self-report surveys, sleep diaries, and actigraphy. Nat Sci Sleep. 2013;5:23–26. doi:10.2147/NSS.S38369. 23620690 PMC3630985

[24] Potocki L , Glaze D, Tan DX, et al. Circadian rhythm abnormalities of melatonin in Smith-Magenis syndrome. J Med Genet. 2000;37(6):428–433. doi:10.1136/jmg.37.6.428. 10851253 PMC1734604

[25] Smith ACM , Morse RS, Introne W, Duncan WC Jr, Duncan WC. Twenty-four-hour motor activity and body temperature patterns suggest altered central circadian timekeeping in Smith–Magenis syndrome, a neurodevelopmental disorder. Am J Med Genet A. 2019;179(2):224–236. doi:10.1002/ajmg.a.61003. 30690916 PMC6699156

[26] Ohayon MM , Vecchierini MF. Normative sleep data, cognitive function and daily living activities in older adults in the community. Sleep. 2005;28(8):981–989. doi:10.1093/sleep/28.8.981. 16218081

[27] Hagenauer MH , Perryman JI, Lee TM, Carskadon MA. Adolescent changes in the homeostatic and circadian regulation of sleep. DNE. 2009;31(4):276–284. doi:10.1159/000216538. PMC2820578 19546564

[28] Falco M , Amabile S, Acquaviva F. RAI1 gene mutations: mechanisms of Smith–Magenis syndrome. Appl Clin Genet. 2017;10:85–94. doi:10.2147/TACG.S128455. 29138588 PMC5680963

[29] Boudreau EA , Johnson KP, Jackman AR, et al. Review of disrupted sleep patterns in Smith–Magenis syndrome and normal melatonin secretion in a patient with an atypical interstitial 17p11.2 deletion. Am J Med Genet A. 2009;149A(7):1382–1391. doi:10.1002/ajmg.a.32846. 19530184 PMC2760428

[30] Micheletti S , Palestra F, Martelli P, et al. Neurodevelopmental profile in Angelman syndrome: more than low intelligence quotient. Ital J Pediatr. 2016;42(1):91. doi:10.1186/s13052-016-0301-4. 27769316 PMC5073425

[31] Wolters PL , Gropman AL, Martin SC, et al. Neurodevelopment of children under 3 years of age with Smith-Magenis syndrome. Pediatr Neurol. 2009;41(4):250–258. doi:10.1016/j.pediatrneurol.2009.04.015. 19748044 PMC2785222

[32] Mindell JA , Owens JA. A Clinical Guide to Pediatric Sleep: Diagnosis and Management of Sleep Problems. Lippincott Williams & Wilkins; 2015.

[33] Smith PL , Little DR. Small is beautiful: in defense of the small-N design. Psychon Bull Rev. 2018;25(6):2083–2101. doi:10.3758/s13423-018-1451-8. 29557067 PMC6267527

[34] Crowley SJ , Acebo C, Carskadon MA. Sleep, circadian rhythms, and delayed phase in adolescence. Sleep Med. 2007;8(6):602–612. doi:10.1016/j.sleep.2006.12.002. 17383934

[35] Ohayon MM , Carskadon MA, Guilleminault C, Vitiello MV. Meta-analysis of quantitative sleep parameters from childhood to old age in healthy individuals: developing normative sleep values across the human lifespan. Sleep. 2004;27(7):1255–1273. doi:10.1093/sleep/27.7.1255. 15586779

[36] Jan JE , Owens JA, Weiss MD, et al. Sleep hygiene for children with neurodevelopmental disabilities. Pediatrics. 2008;122(6):1343–1350. doi:10.1542/peds.2007-3308. 19047255

[37] Allanson JE , Greenberg F, Smith ACM. The face of Smith-Magenis syndrome: a subjective and objective study. J Med Genet. 1999;36(5):394–397. doi:10.1136/jmg.36.5.394. 10353786 PMC1734375

[38] Agar G , Oliver C, Richards C. Direct assessment of overnight parent-child proximity in children with behavioral insomnia: extending models of operant and classical conditioning. Behav Sleep Med. 2022;21(0):254–272. doi:10.1080/15402002.2022.2076681. 35796281

[39] Didden R , Sigafoos J, Lancioni GE. Chapter 26 - unmodified extinction for childhood sleep disturbance. In: Perlis M, Aloia M, Kuhn B, eds. Behavioral Treatments for Sleep Disorders. Practical Resources for the Mental Health Professional. Academic Press; 2011:257–263. doi:10.1016/B978-0-12-381522-4.00026-2

[40] Fujiwara Y , Arakawa T, Fass R. Gastroesophageal reflux disease and sleep disturbances. J Gastroenterol. 2012;47(7):760–769. doi:10.1007/s00535-012-0601-4. 22592763

[41] Long AC , Krishnamurthy V, Palermo TM. Sleep disturbances in school-age children with chronic pain. J Pediatr Psychol. 2008;33(3):258–268. doi:10.1093/jpepsy/jsm129. 18079168 PMC2824539

[42] Agar G , Oliver C, Trickett J, Licence L, Richards C. Sleep disorders in children with Angelman and Smith-Magenis syndromes: the assessment of potential causes of disrupted settling and night time waking. Res Dev Disabil. 2020;97:103555. doi:10.1016/j.ridd.2019.103555. 31838315

[43] Agostini A , Pignata S, Camporeale R, et al. Changes in growth and sleep across school nights, weekends and a winter holiday period in two Australian schools. Chronobiol Int. 2018;35(5):691–704. doi:10.1080/07420528.2018.1430037. 29372811

[44] Hansen M , Janssen I, Schiff A, Zee PC, Dubocovich ML. The impact of school daily schedule on adolescent sleep. Pediatrics. 2005;115(6):1555–1561. doi:10.1542/peds.2004-1649. 15930216

[45] Acebo C , Sadeh A, Seifer R, et al. Estimating sleep patterns with activity monitoring in children and adolescents: how many nights are necessary for reliable measures? Sleep. 1999;22(1):95–103. doi:10.1093/sleep/22.1.95. 9989370

[46] Berger AM , Wielgus KK, Young-McCaughan S, Fischer P, Farr L, Lee KA. Methodological challenges when using actigraphy in research. J Pain Symptom Manage. 2008;36(2):191–199. doi:10.1016/j.jpainsymman.2007.10.008. 18400460 PMC2542506

[47] Koo TK , Li MY. A Guideline of selecting and reporting intraclass correlation coefficients for reliability research. J Chiropr Med. 2016;15(2):155–163. doi:10.1016/j.jcm.2016.02.012. 27330520 PMC4913118

[48] Sparrow SS , Cicchetti DV, Balla DA. *Vineland Adaptive Behavior Scales – Second Edition (Vineland–II)* . Published 2005. https://psycnet.apa.org/doiLanding?doi=10.1037%2Ft15164-000

[49] Simonds JF , Parraga H. Prevalence of sleep disorders and sleep behaviors in children and adolescents. J Am Acad Child Adolesc Psychiatry. 1982;21:383–388. doi:10.1016/s0002-7138(09)60942-0. 6981663

[50] Wiggs L , Stores G. Severe sleep disturbance and daytime challenging behaviour in children with severe learning disabilities. J Intellect Disabil Res. 1996;40(Pt 6):518–528. doi:10.1046/j.1365-2788.1996.799799.x. 9004112

[51] Johnson CR , Turner KS, Foldes EL, Malow BA, Wiggs L. Comparison of sleep questionnaires in the assessment of sleep disturbances in children with autism spectrum disorders. Sleep Med. 2012;13(7):795–801. doi:10.1016/j.sleep.2012.03.005. 22609024 PMC3398235

[52] Owens JA , Spirito A, McGuinn M. The Children’s Sleep Habits Questionnaire (CSHQ): psychometric properties of a survey instrument for school-aged children. Sleep. 2000;23(8):1–9. doi:10.1093/sleep/23.8.1d. 11145319

[53] Malow BA , Crowe C, Henderson L, et al. A sleep habits questionnaire for children with autism spectrum disorders. J Child Neurol. 2009;24(1):19–24. doi:10.1177/0883073808321044. 19168814

[54] Breau LM , Camfield CS, McGrath PJ, Finley GA. Risk factors for pain in children with severe cognitive impairments. Dev Med Child Neurol. 2004;46(6):364–371. doi:10.1017/s001216220400060x. 15174527

[55] Breau LM , McGrath PJ, Camfield CS, Finley GA. Psychometric properties of the non-communicating children’s pain checklist-revised. Pain. 2002;99(1):349–357. doi:10.1016/s0304-3959(02)00179-3. 12237214

[56] Berg K , Arron K, Burbidge C, Moss J, Oliver C. Carer-reported contemporary health problems in people with severe and profound intellectual disability and genetic syndromes. J Policy Pract Intell Disabilities. 2007;4(2):120–128. doi:10.1111/j.1741-1130.2007.00109.x.

[57] Waite J , Heald M, Wilde L, et al. The importance of understanding the behavioural phenotypes of genetic syndromes associated with intellectual disability. Paed Child Health. 2014;24(10):468–472. doi:10.1016/j.paed.2014.05.002.

[58] Eden KE , de Vries PJ, Moss J, Richards C, Oliver C. Self-injury and aggression in tuberous sclerosis complex: cross syndrome comparison and associated risk markers. J Neurodevelop Disord. 2014;6(1):10. doi:10.1186/1866-1955-6-10. PMC4017702 24822087

[59] Symons FJ , Harper VN, McGrath PJ, Breau LM, Bodfish JW. Evidence of increased non-verbal behavioral signs of pain in adults with neurodevelopmental disorders and chronic self-injury. Res Dev Disabil. 2009;30(3):521–528. doi:10.1016/j.ridd.2008.07.012. 18789843 PMC3533421

[60] Burbidge C , Oliver C, Moss J, et al. The association between repetitive behaviours, impulsivity and hyperactivity in people with intellectual disability. J Intellect Disabil Res. 2010;54(12):1078–1092. doi:10.1111/j.1365-2788.2010.01338.x. 20977516

[61] Spilsbury JC , Storfer-Isser A, Drotar D, Rosen CL, Kirchner HL, Redline S. Effects of the home environment on school-aged children’s sleep. Sleep. 2005;28(11):1419–1427. doi:10.1093/sleep/28.11.1419. 16335483

[62] Jeffreys H. The Theory of Probability. OUP Oxford; 1998.

[63] Surtees ADR , Richards C, Clarkson EL, et al. Sleep problems in autism spectrum disorders: a comparison to sleep in typically developing children using actigraphy, diaries and questionnaires. Res Autism Spectrum Disord. 2019;67:101439. doi:10.1016/j.rasd.2019.101439.

[64] Morley S , Dowzer C. Manual for the leeds reliable change indicator: simple excel® applications for the analysis of individual patient and group data. University of Leeds, Leeds, UK 2014;13.

[65] Oliver C , Royston R, Crawford H, et al. Informant Assessments of Behaviour and Affect for People with Intellectual Disability; 2019.

[66] Maas APHM , Didden R, Korzilius H, et al. Psychometric properties of a sleep questionnaire for use in individuals with intellectual disabilities. Res Dev Disabil. 2011;32(6):2467–2479. doi:10.1016/j.ridd.2011.07.013. 21840166

[67] Pandi-Perumal SR , Spence DW, BaHammam AS. Polysomnography: an overview. In: Pagel JF, Pandi-Perumal SR, eds. Primary Care Sleep Medicine: A Practical Guide. Springer; 2014:29–42. doi:10.1007/978-1-4939-1185-1_4.

[68] Paasch V , Hoosier TM, Accardo J, Ewen JB, Slifer KJ. Technical tips: performing EEGs and polysomnograms on children with neurodevelopmental disabilities. Neurodiagnostic J. 2012;52(4):333–348. PMC3704171 23301283

