# Supplementary material for: The developmental trajectory of sleep in children with Smith-Magenis syndrome compared to typically developing peers: a 3-year follow-up study
Source: Sleep Adv. 2023 Sep 8;4(1):zpad034. doi: 10.1093/sleepadvances/zpad034 (PMC10559836; doi:10.1093/sleepadvances/zpad034)
Supplement: zpad034_suppl_Supplementary_Materials [file zpad034_suppl_supplementary_materials.docx]

The developmental trajectory of sleep in children with Smith-Magenis syndrome compared to typically developing peers: A three-year follow-up study

**Supplementary Material**

Dr Georgie Agar^a,b^, Professor Chris Oliver^b^, Dr Jayne Spiller^b,c^ & Dr Caroline Richards^b,d^

^a^School of Psychology, Aston University, UK

^b^School of Psychology, University of Birmingham, UK

^c^School of Psychology and Vision Sciences, University of Leicester, UK

^d^Cerebra Network for Neurodevelopmental Disorders, Birmingham, UK

Corresponding author: Dr Georgie Agar, School of Psychology, College of Health and Life Sciences, Aston Triangle Birmingham, B4 7ET, UK. Email: [g.agar@aston.ac.uk](mailto:g.agar@aston.ac.uk)

# Supplementary Material 1: Recruitment and Retention

#
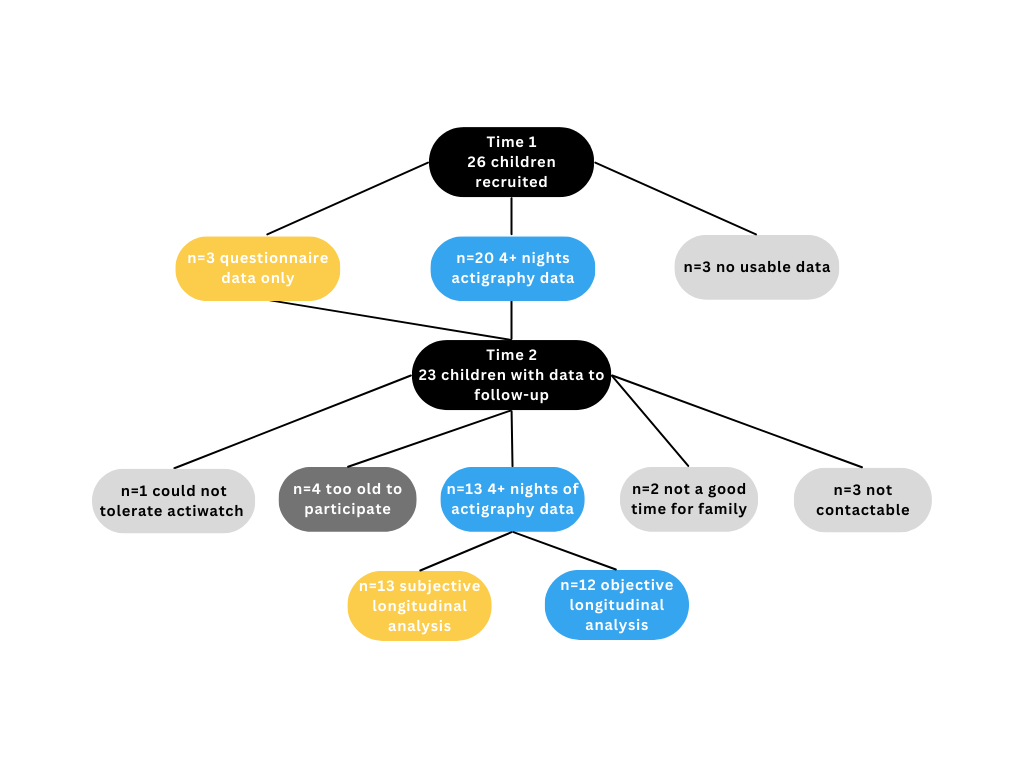


*Figure 1.*Summary of the process of recruitment and retention for children with Smith-Magenis syndrome at Time 1 and Time 2. Grey boxes indicate the number of children who did not provide usable data at each time point for various reasons, including 4 children who had exceeded the age of 15 by Time 2 and were therefore not eligible to take part due to ethical approvals. Blue boxes indicate the number of children who provided at least 4 nights of usable actigraphy data at each time point. Yellow boxes indicate the number of children for whom informant-based sleep assessments were available at each time point.


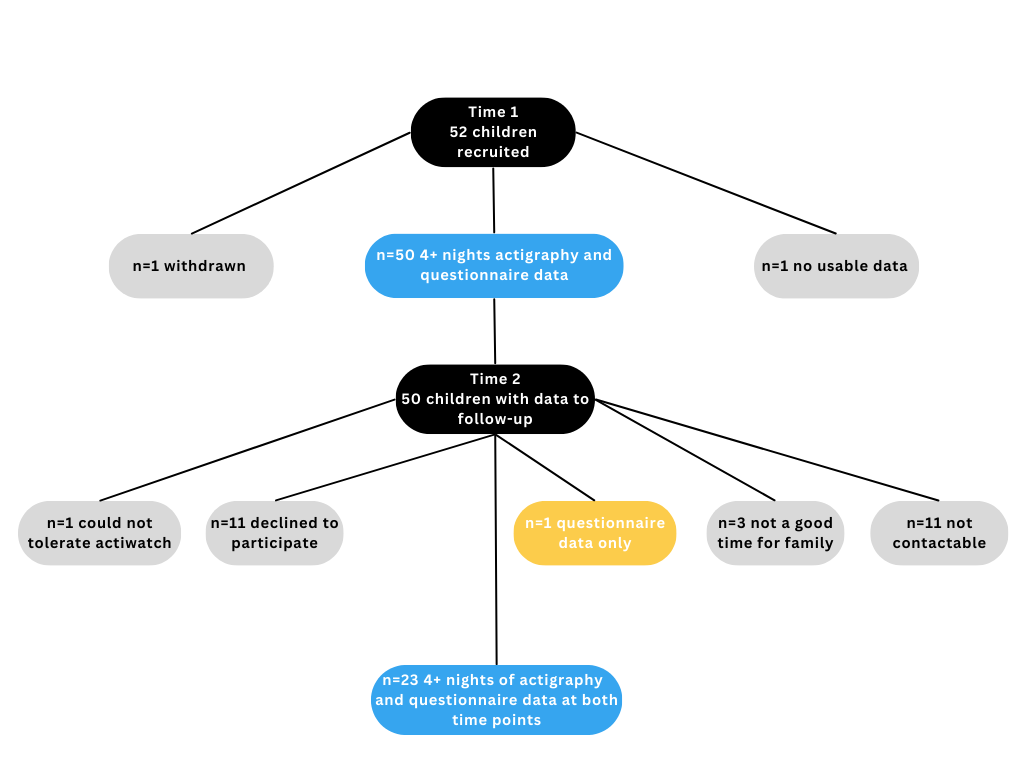


*Figure 2.* Summary of the process of recruitment and retention for typically developing at Time 1 and Time 2. Grey boxes indicate the number of children who did not provide usable data at each time point for various reasons. Blue boxes indicate the number of children who provided at least 4 nights of usable actigraphy data at each time point. Yellow boxes indicate the number of children for whom informant-based sleep assessments were available at each time point.

# Supplementary Material 2: Finalised Matching Strategy (Prioritising Age)

| **SMS** |  |  | **TD** |  |  |
| --- | --- | --- | --- | --- | --- |
| Sex | Exact Age |  | Sex | Exact Age |  |
| Female | 8.994521 |  | Male | 7.128767 |  |
| Male | 9.032877 |  | Female | 7.438356 |  |
| Male | 9.939726 |  | Female | 7.6 |  |
| Female | 10.38082 |  | Male | 7.728767 |  |
| Female | 10.47671 |  | Female | 7.909589 |  |
| Male | 10.67123 |  | Male | 8.476712 |  |
| Male | 10.87123 |  | Female | 8.641096 |  |
| Female | 10.99452 |  | Male | 9.147945 |  |
| Male | 11.75616 |  | Male | 9.490411 |  |
| Female | 11.8411 |  | Male | 9.539726 |  |
| Female | 12.07123 |  | Male | 9.558904 |  |
| Female | 12.21096 |  | Male | 9.682192 |  |
| Male | 14.98904 |  | Male | 9.731507 |  |
|  |  |  | Male | 10.03014 |  |
|  |  |  | Male | 10.54247 |  |
|  |  |  | Male | 10.92877 |  |
|  |  |  | Female | 11.46301 |  |
|  |  |  | Male | 12.03836 |  |
|  |  |  | Male | 12.15342 |  |
|  |  |  | Female | 12.3726 |  |
|  |  |  | Male | 12.72329 |  |
|  |  |  | Female | 13.72603 |  |
|  |  |  | Female | 15.19452 |  |
|  |  |  | Male | 15.69589 |  |
|  |  |  | Male | 17.73699 |  |

# Supplementary Material 3: Actigraphy Cleaning Protocol

Please use this reference when citing this work:

Trickett J., Heald, M., Surtees, A., Clarkson, E., Agar, G., Oliver, C., & Richards, C. (2017). *Actigraphy Cleaning Protocol*. University of Birmingham, Birmingham.

**Actigraphy Cleaning Protocol**

Jayne Trickett, Mary Heald, Andrew Surtees, Emma Clarkson, Georgie Agar, Chris Oliver and Caroline Richards

*Cerebra Network for Neurodevelopmental Disorders*

*School of Psychology*

*University of Birmingham*

**Data cleaning**

**Needed to complete cleaning:**

1. Open file on Philips Actiware
2. Child diary.

**Step-1**: Exclude any automatically-coded intervals which occur after the watch has been collected (E, Shift E, Ctrl E). This information can be gained from the sleep diary.


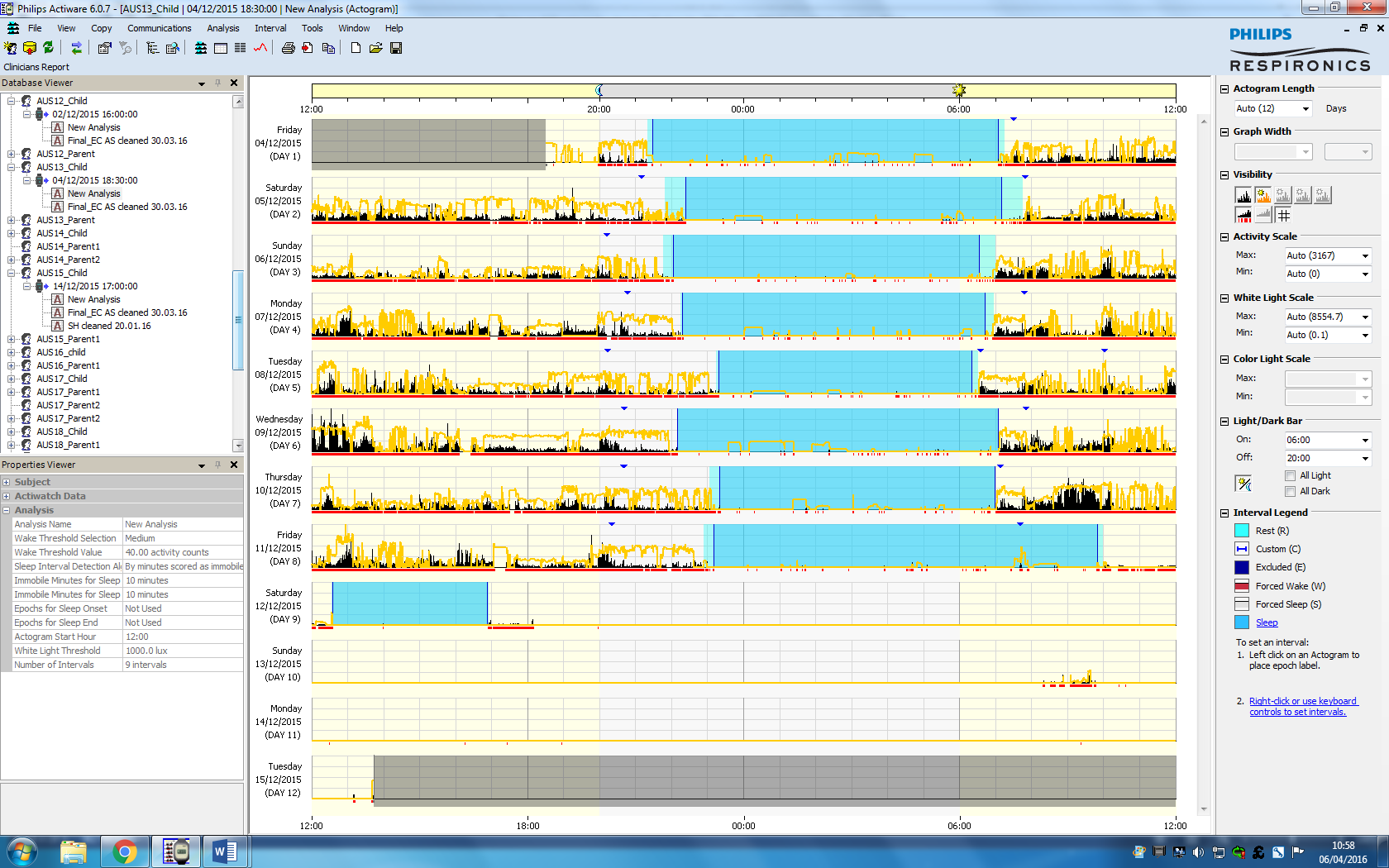


** Automatically-calculated sleep interval occurring on Saturday 12^th^ has taken place after the watch has been collected. This interval has therefore been excluded*

**Step-2**: Exclude any nights during which the parent identifies a time when the watch was taken off.

-
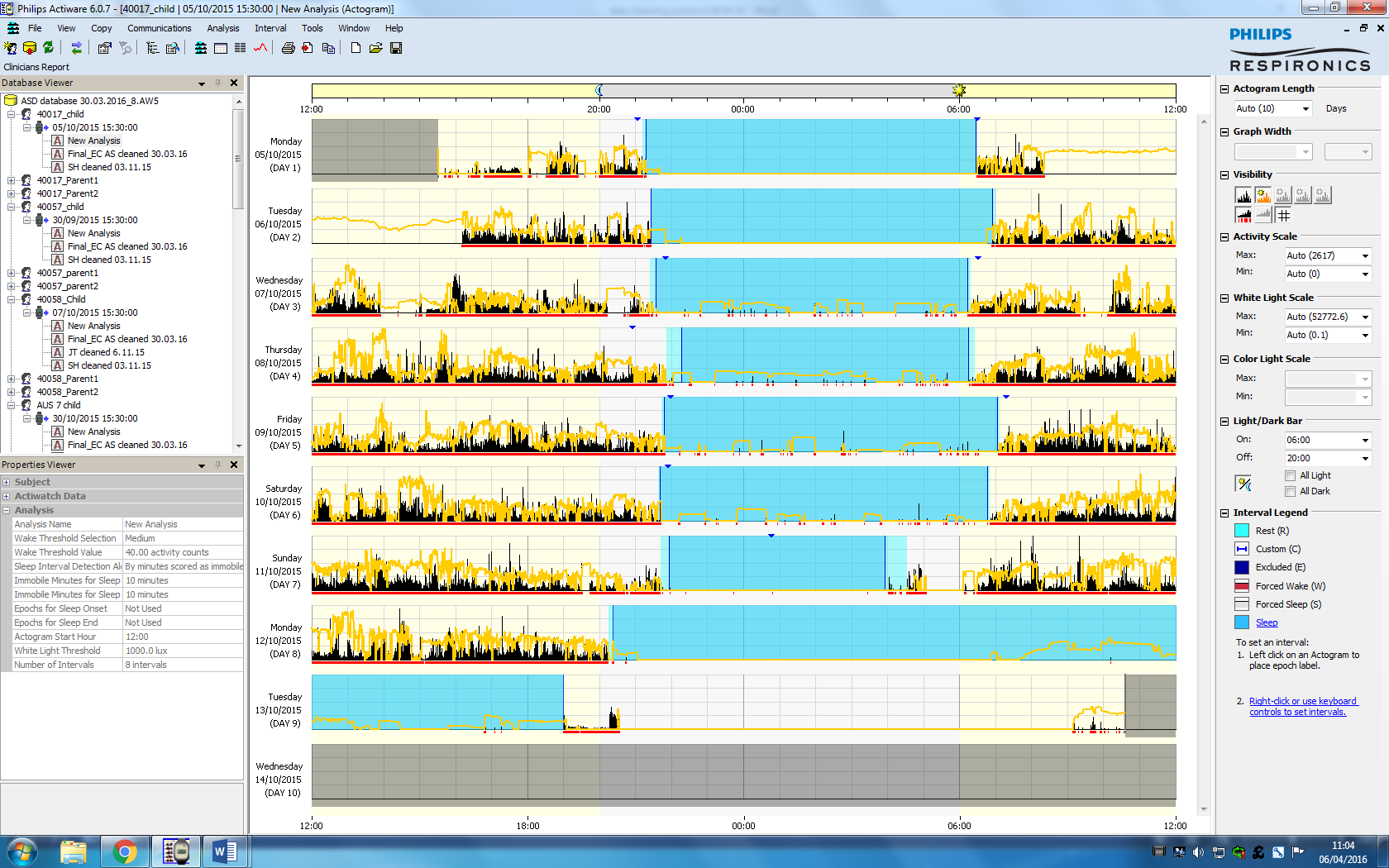
Open child diary to the relevant night, and confirm if parent reports any times that watch was taken off between lights out and wake-up time. If there are any such times check against actogram for consistency (i.e. no movement at this time). If consistent, exclude this night from data in actogram (E, Shift E, Ctrl E). If parent reports that the watch has been removed, but this is not evident on the actogram (i.e. evidence of movement during this time), keep the existing interval as it is and continue to step 3.

** Parent has recorded that the watch was removed on the night of Monday 6^th^. Inspection of the actogram suggests that there was no evidence of activity, and therefore appears to be consistent with parent report. The interval has therefore been excluded.*


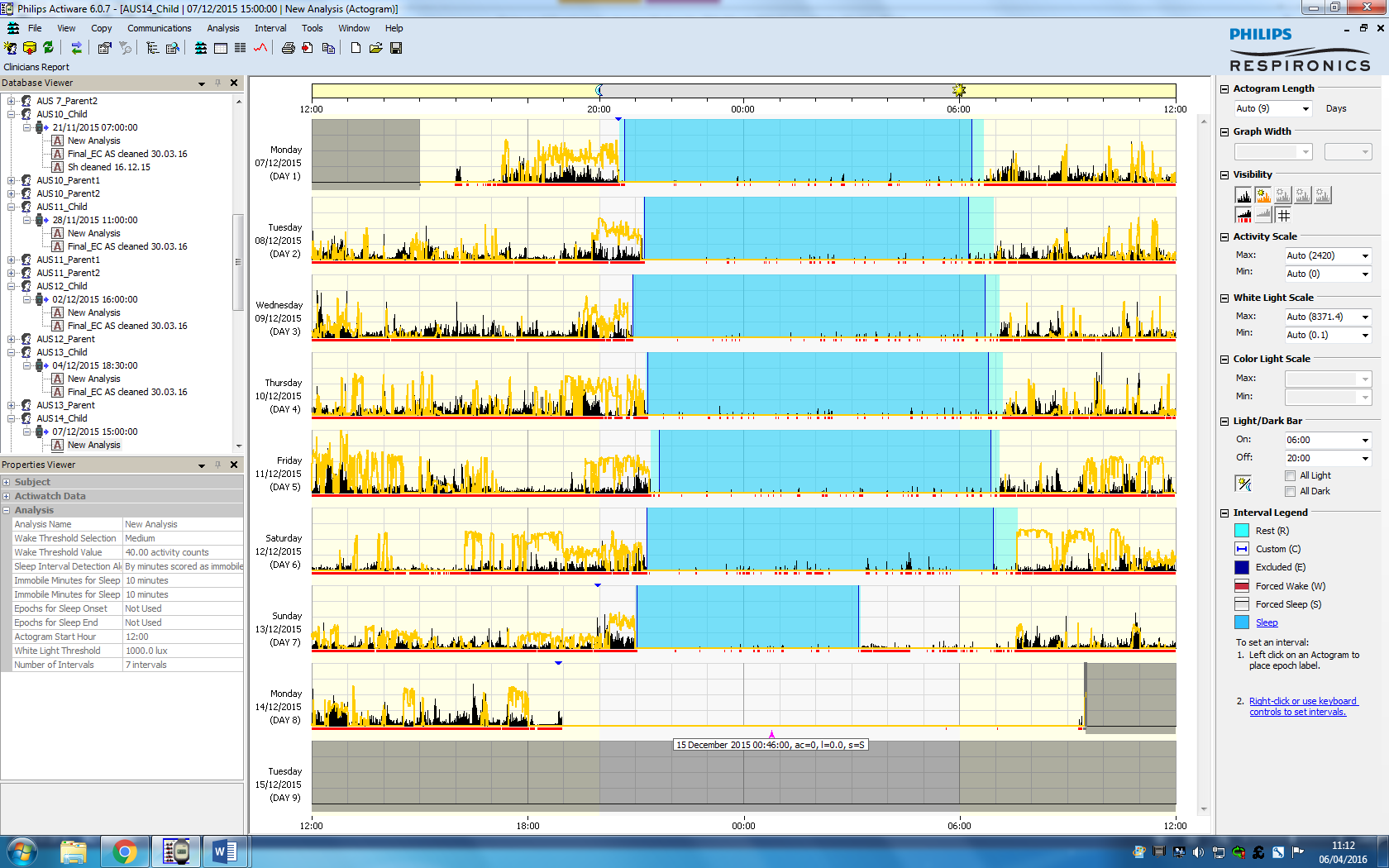


** Parent has recorded that the watch was removed for part of the night of Thursday 10^th^. Inspection of the actogram suggests that there was activity throughout the interval, and this therefore appears to be inconsistent with parent report. The automatically-calculated interval has therefore been left.*

**Step-3**: Exclude any nights during which the watch appears to have been taken off, but this was not noted in parent diary.

- Visually inspect each night on actogram. If on any night, there is no recorded activity (**0 in activity column** of data list) for a period of 2 hours or more, exclude this whole night from actogram (E, Shift E, Ctrl E).


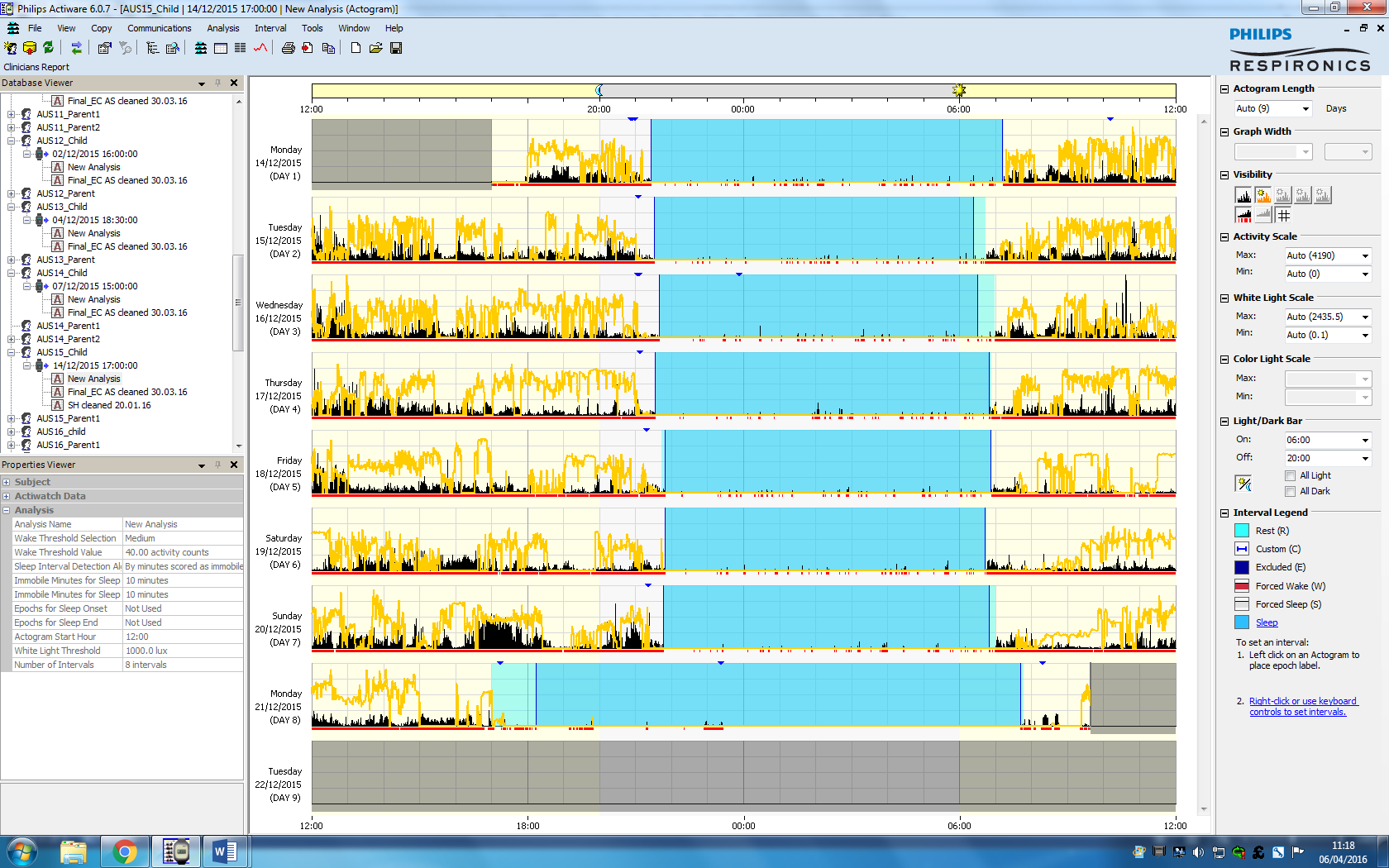


** Parent has not recorded that the watch was removed during the night of Monday 21^st^, but inspection of the actogram suggests that there were periods greater than 2hours without any evidence of activity. This would suggest that the watch was removed, and the automatically-calculated interval has therefore been excluded.*

**Step-4**: Clear any automatically-calculated sleep intervals from the day time and insert interval to night time

- Note any occasions on which the software has coded the sleep interval as in the day-time. Criteria for this is if the automatically-coded interval both starts and ends outside of period noted as sleep in parent diary. For any intervals on which this is the case, clear the sleep interval (Right click, clear interval).
- New interval should be inserted.
- To allocate start time of new interval: 1. Find first period of 20 minutes of sleep after lights out in diary (40 epochs coded as **0 in sleep/wake column** in data list). From there, go back to the last period of 10 minutes of activity (20 epochs coded as **1 in sleep/wake column**). Start time is first 0 after this.
- To allocate end time: 1. Find last period of 20 minutes of sleep before wake-up time in diary (40 epochs coded as **0 in sleep/wake column**). From there, go forward to the first period of 10 minutes of activity (20 epochs coded as **1 in sleep/wake column**). End time is first 0 before this.


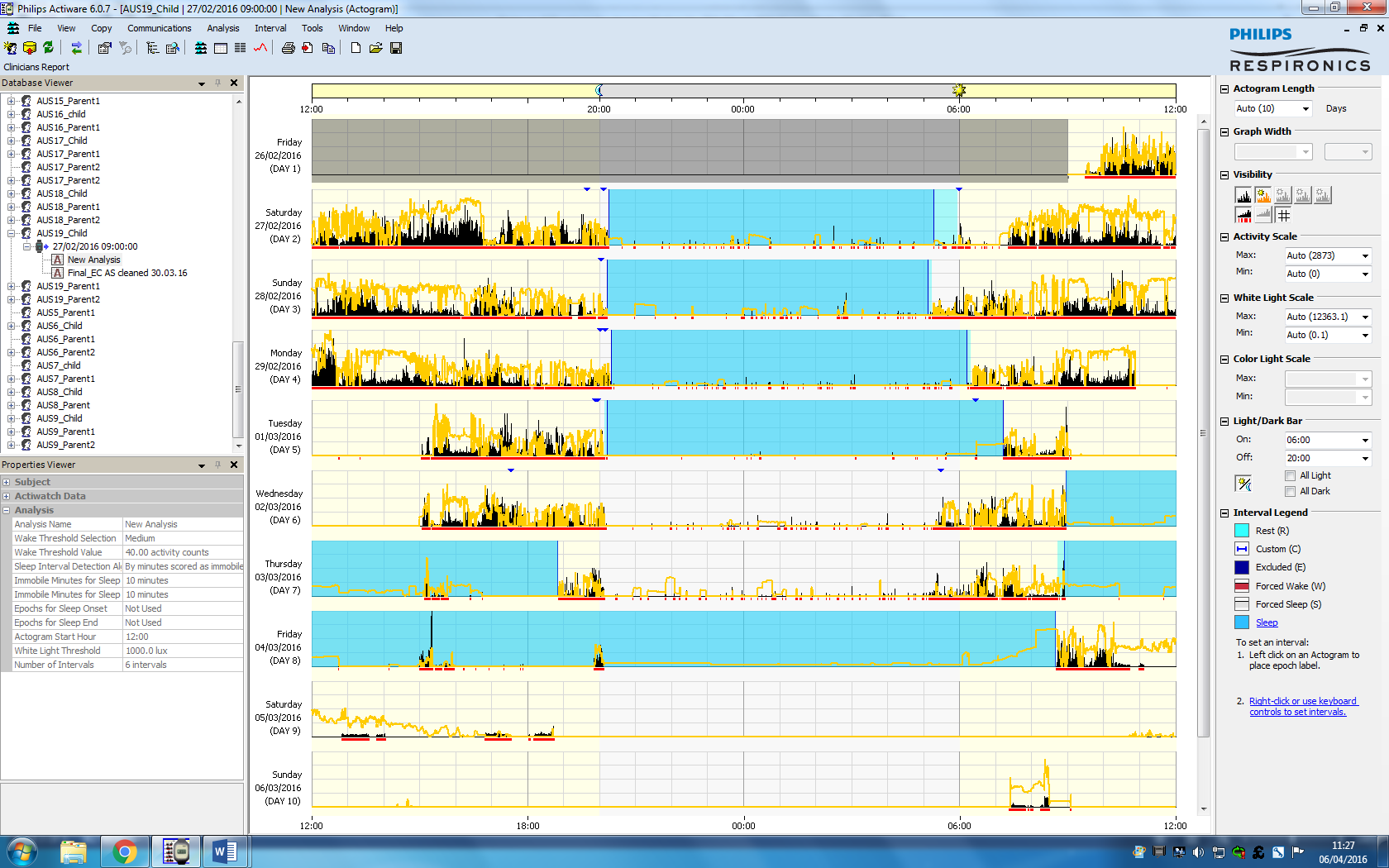

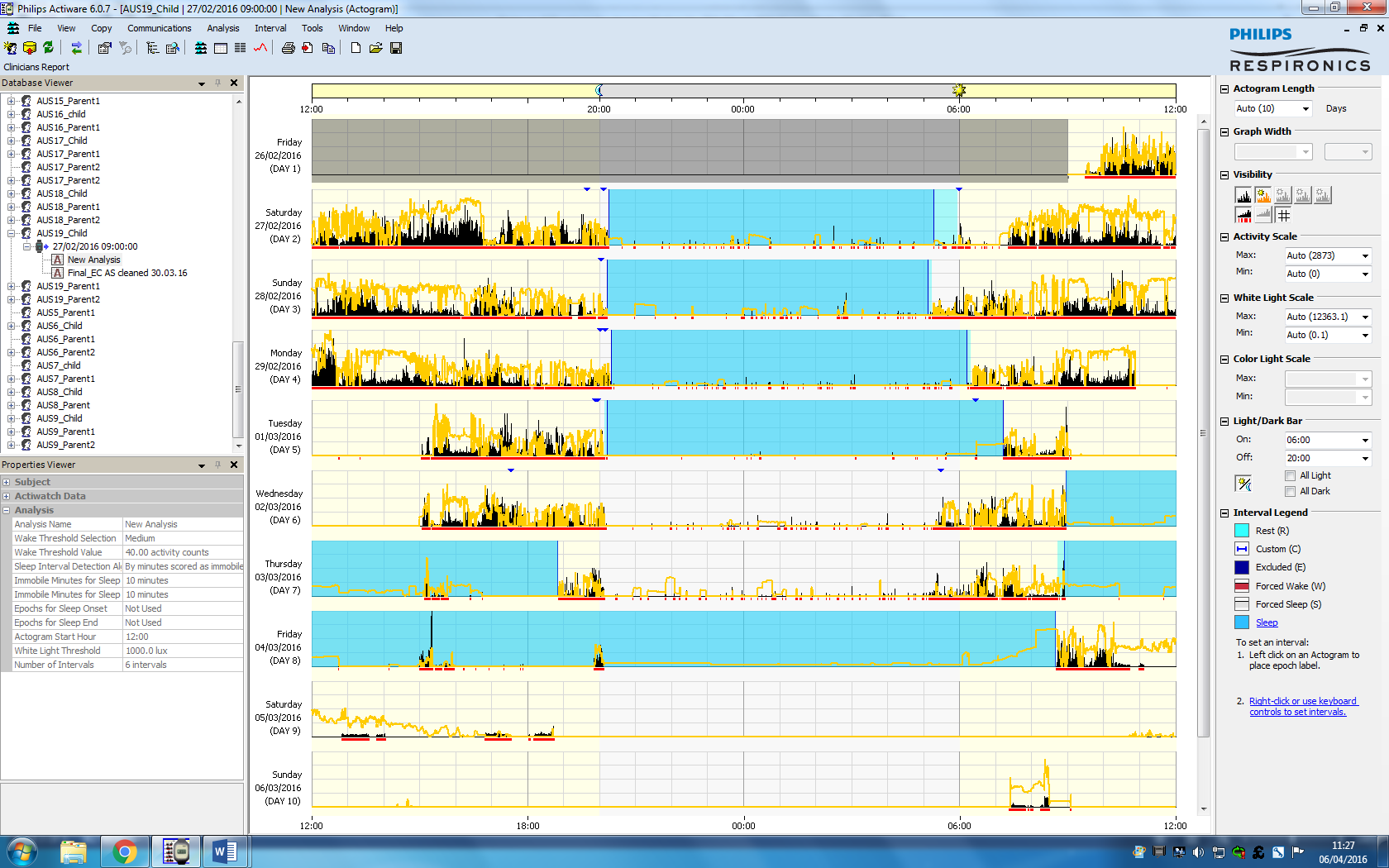


** Sleep interval has been automatically-calculated to fall between 9am and 7pm on Thursday 3^rd^. When consulting the parent diary, it can be seen that this interval falls outside of the time that the parent reported the child to be in bed, and so we can assume that the interval has been calculated incorrectly. As a result, we can clear the incorrect intervals, and insert new intervals.*


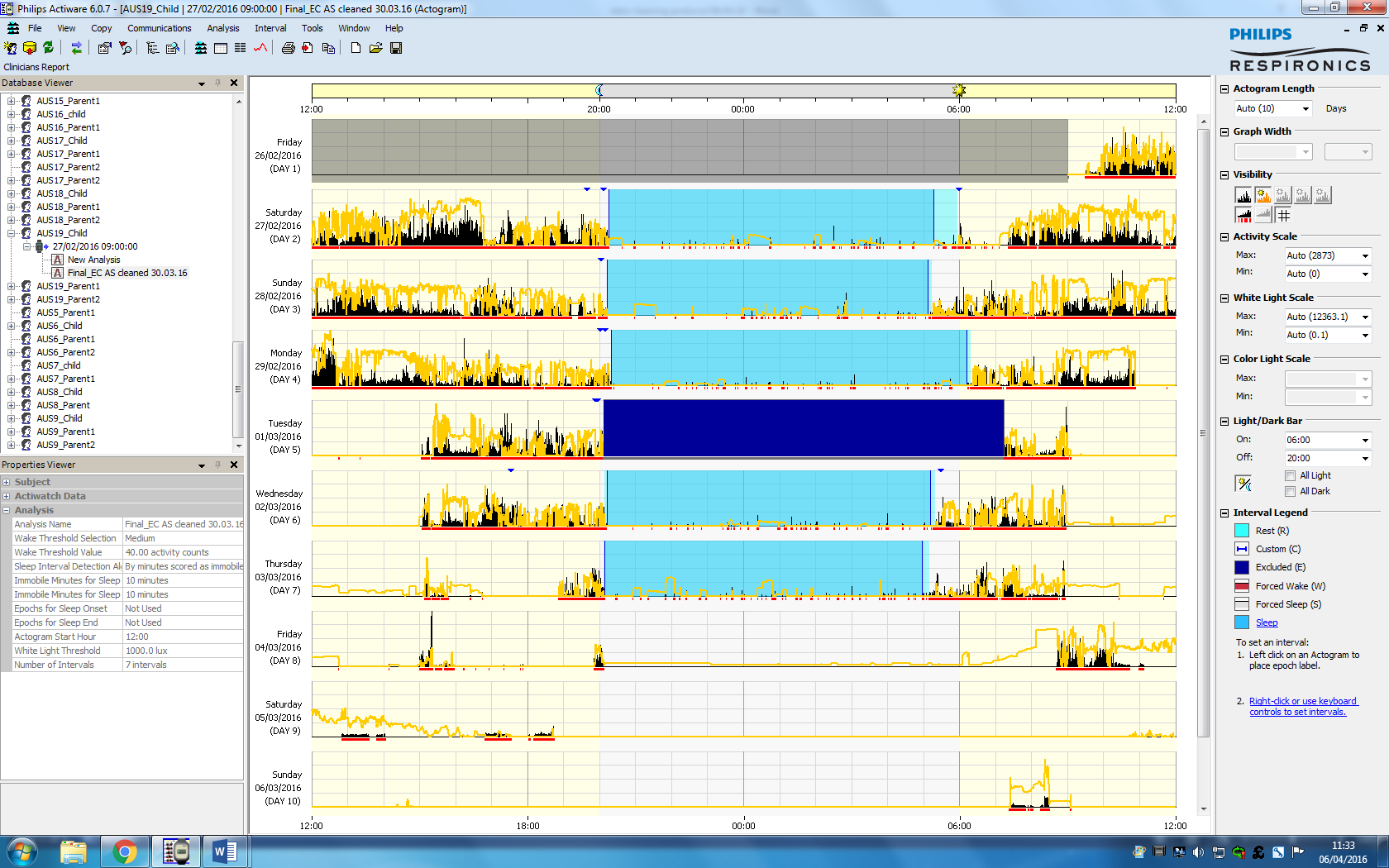

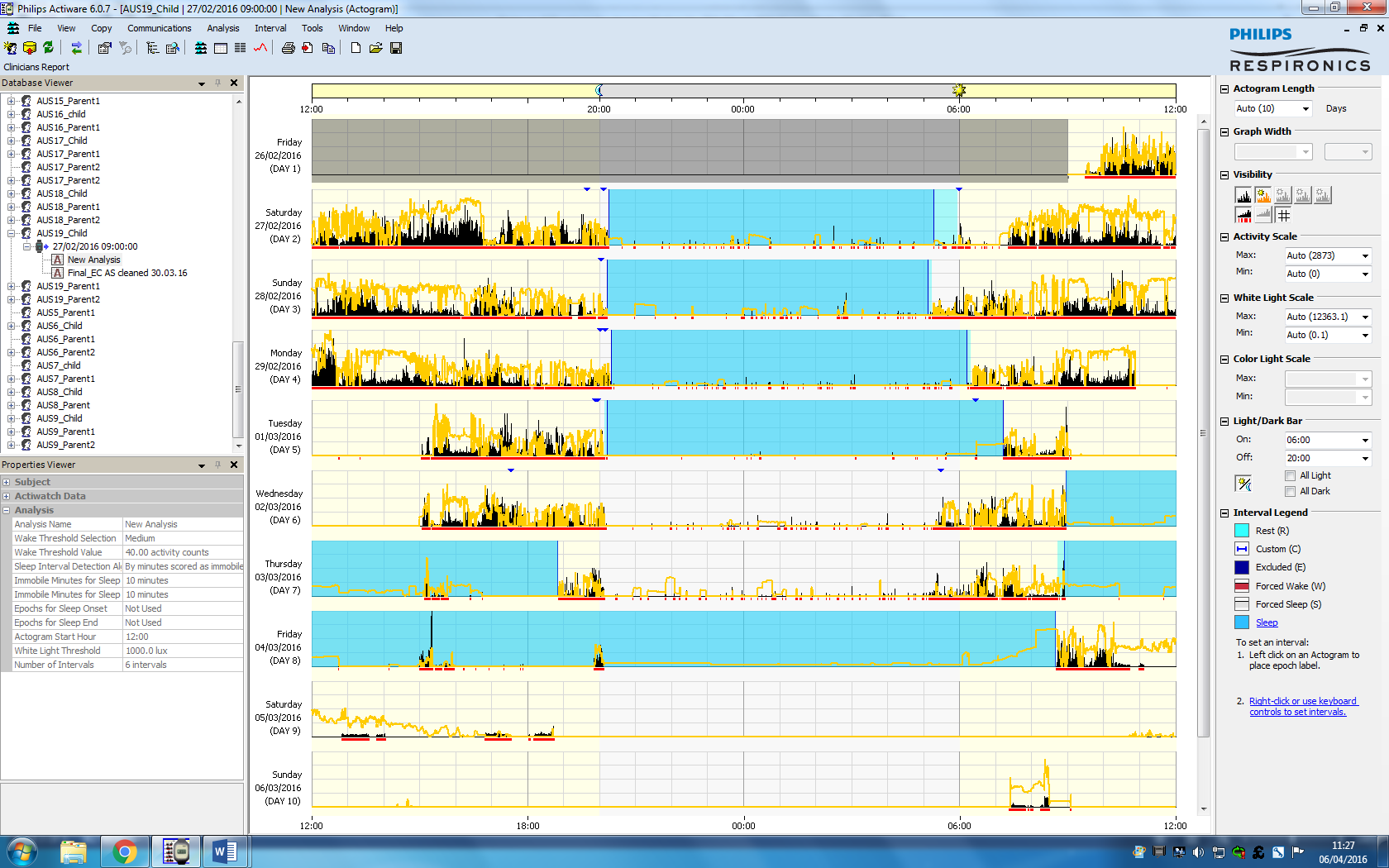


** New sleep intervals have been calculated using the data list, to identify times when the child is likely to have fallen asleep and awoken in the morning based on the data in the sleep/wake column. These data have been used to insert new intervals for Wednesday and Thursday nights.*

**Step-5**: Extend any intervals that have not captured entire night sleep.

- Locate any 20 minute periods coded as sleep in the actogram (40 epochs coded as 0 in sleep/wake column), that are **not** found within the automatically calculated sleep interval, **but are** between lights out and wake-up in sleep diary.
- If period is after the automatically calculated interval, extend interval from sleep period to last point before 10 minutes coded as awake (20 consecutive scores coded as 1 in sleep/wake column on datalist). To do this, clear the original interval and add a new one with the original start time and the new end time.
- If period is before the automatically calculated interval, extend interval from sleep period to first point after 10 minutes coded as awake (20 consecutive epochs coded as 1 in sleep/wake column on datalist). To do this, clear the original interval and add a new one with the original end time and the new start time.


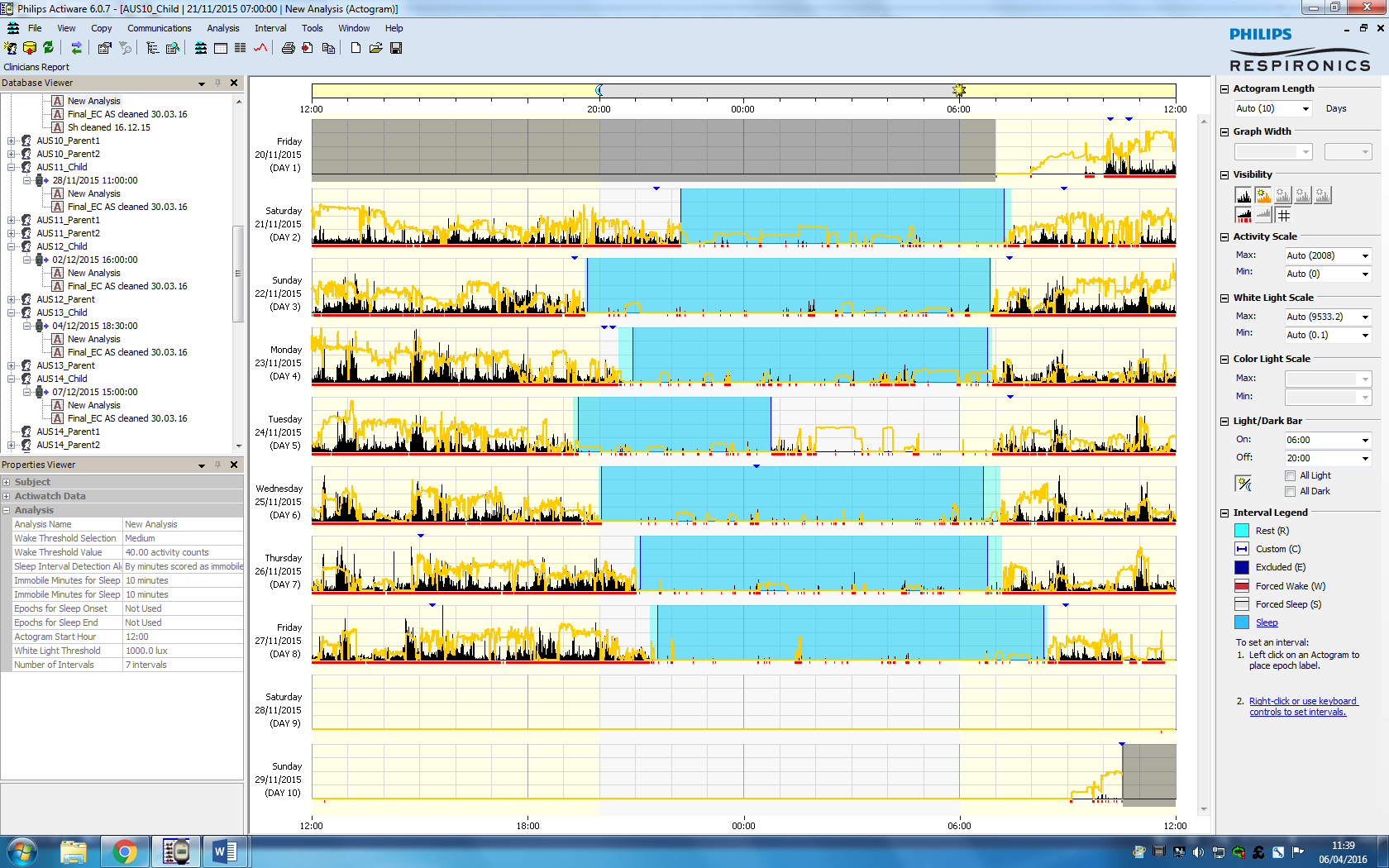

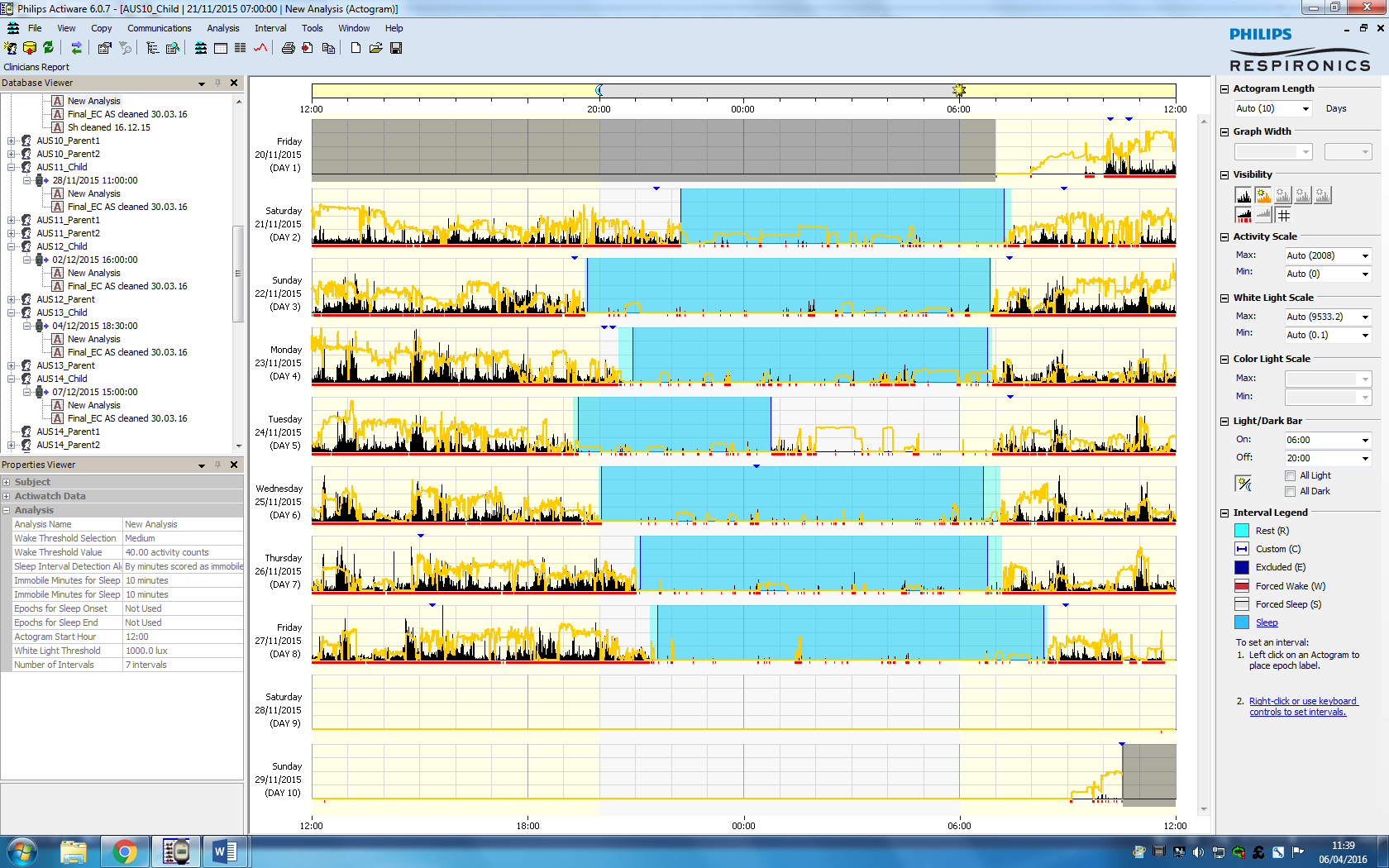


** The automatically-calculated sleep interval suggests that child is awake from 1 am on Wednesday 25^th^. However, inspection of the actogram suggests that there are several periods of non-activity after 1 am. Looking at the actogram more closely, it can be seen that some of these periods are greater than 20 mins, which implies that the child has returned to sleep and the automatically-calculated sleep interval has incorrectly estimated the child’s final waking time as being too early. As a result, we can clear the incorrect interval, and insert a new interval.*


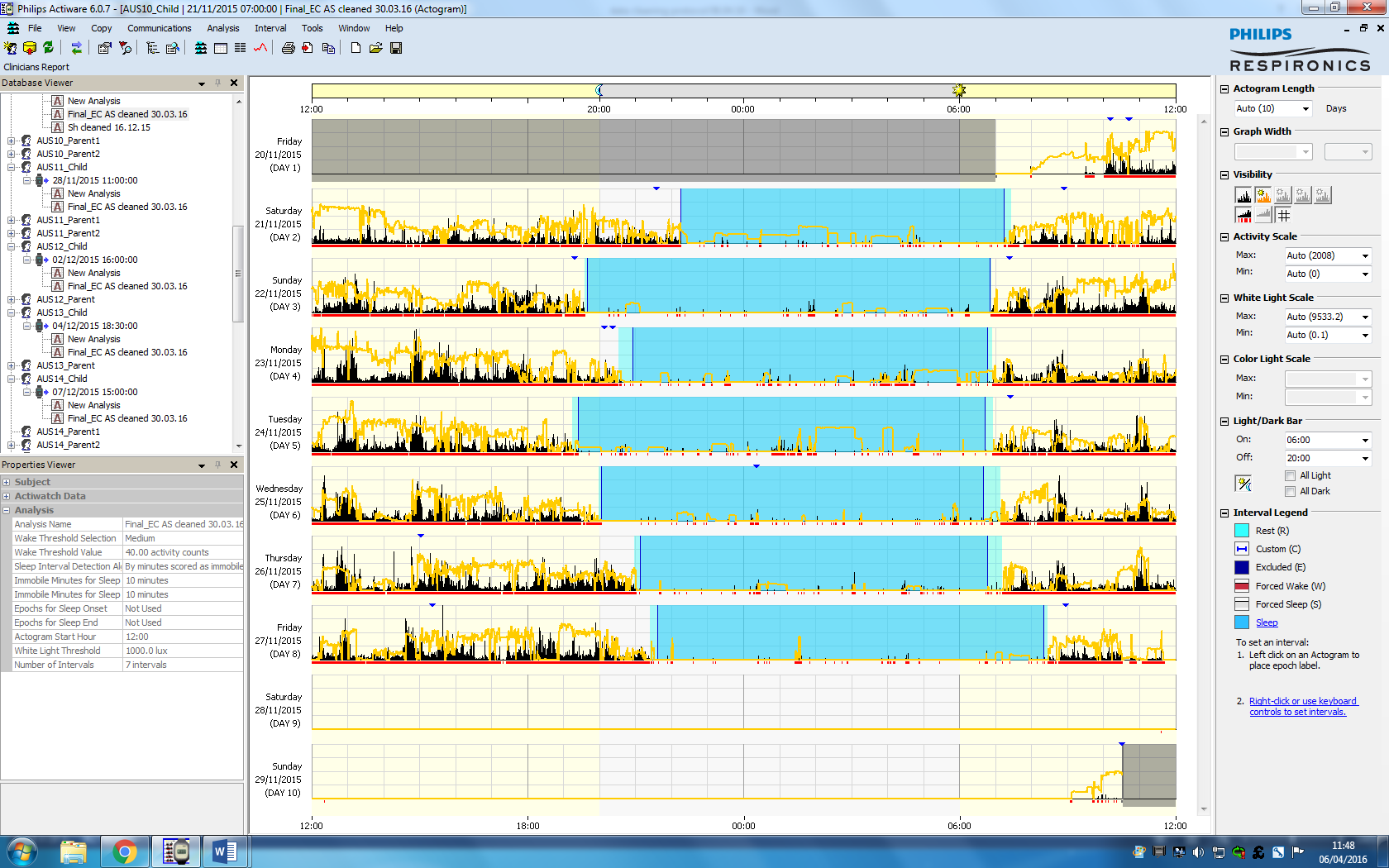

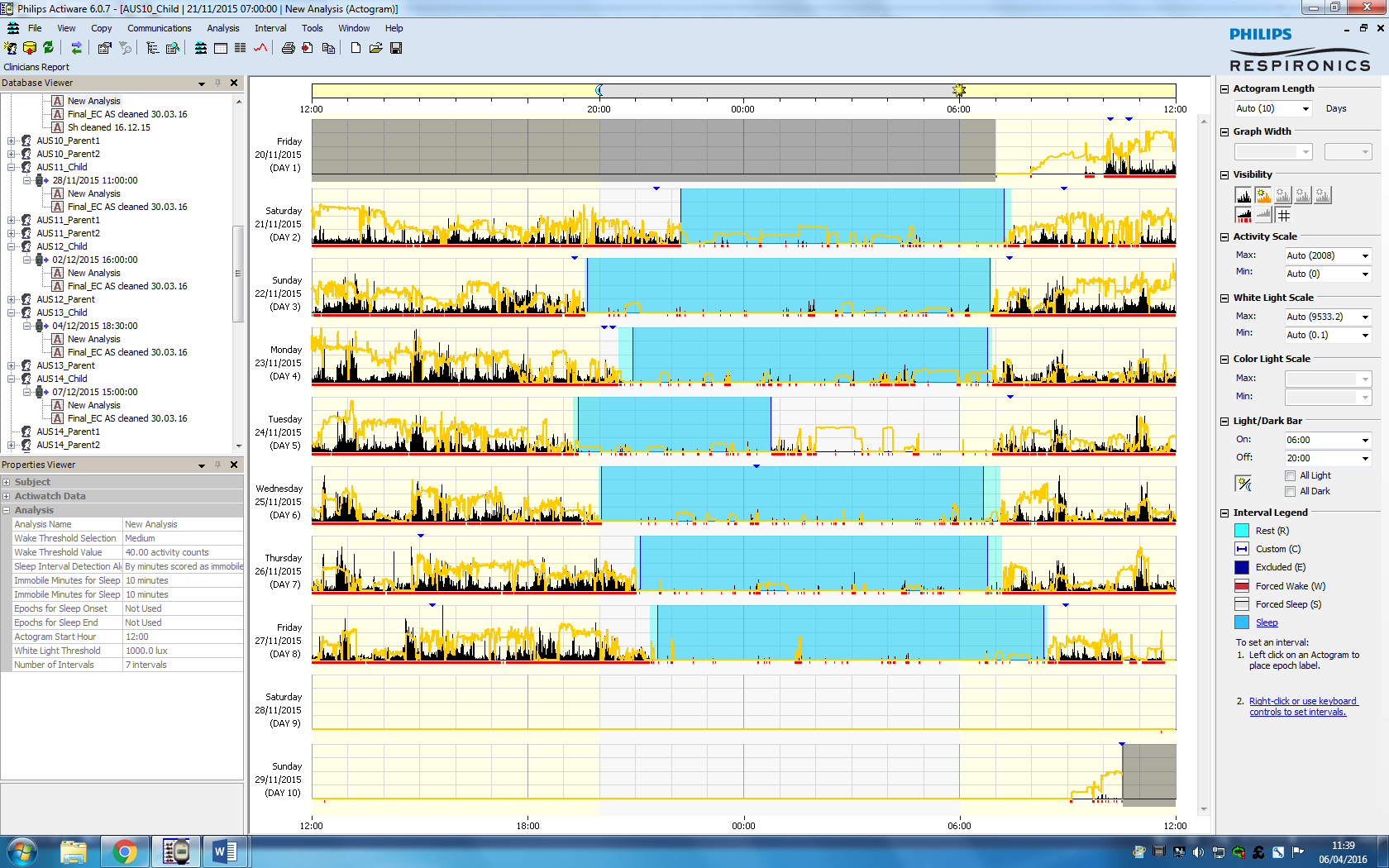


** A new sleep interval has been calculated using the data list, to identify the time at which the child is likely to have awoken in the morning based on the data in the sleep/wake column. The original time at which the sleep interval was automatically estimated to have begun has remained the same. The original start time, and new end time have therefore been used to insert a new interval.*

**Step-6:** Exclude any intervals that have twice the duration of the average Total Sleep Time

- Note any occasion where the software has created a sleep interval where the duration of Total Sleep Time and/or the sleep diary has stated that time between lights out and waking up time is twice that of the average TST. Exclude sleep interval.


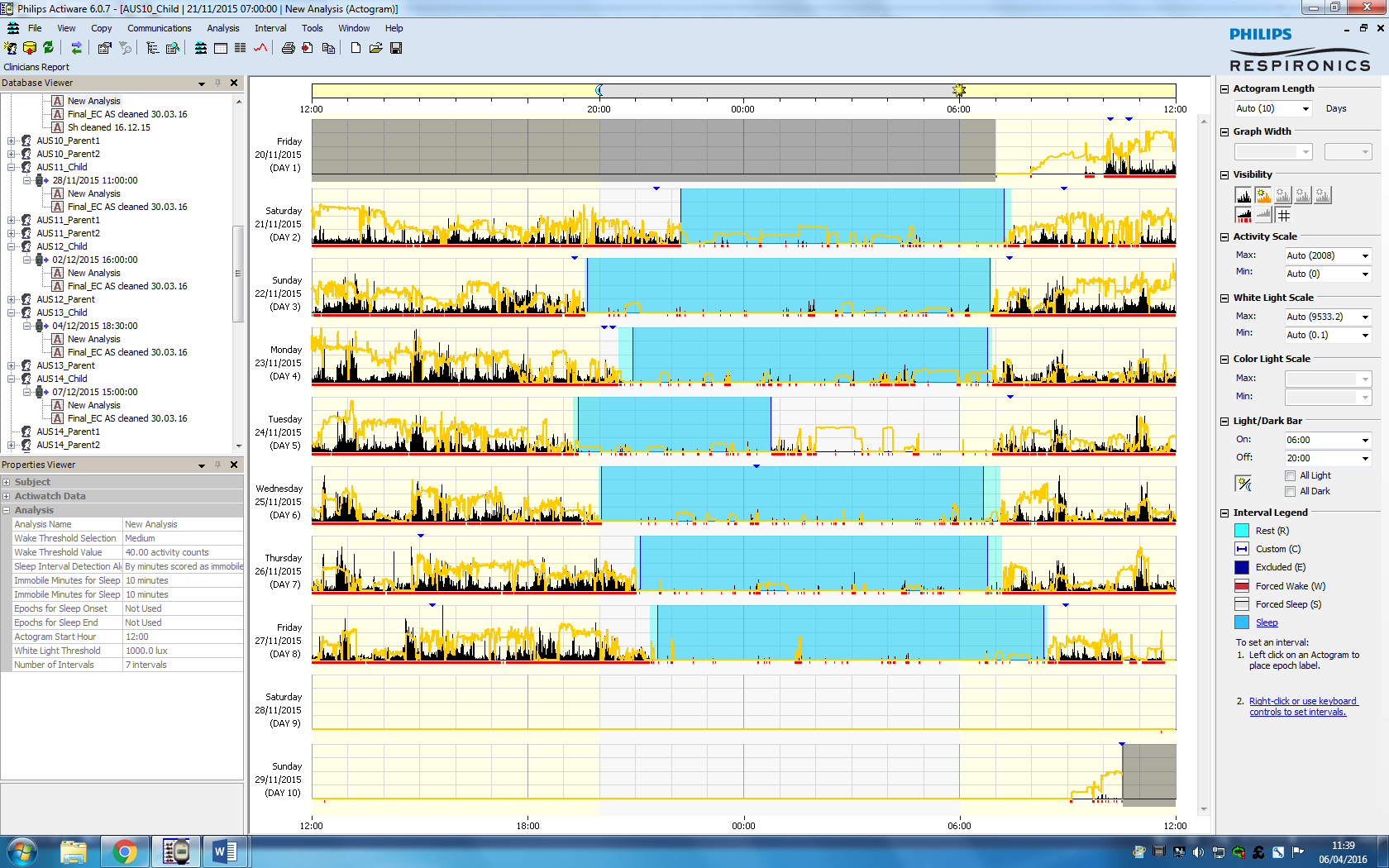

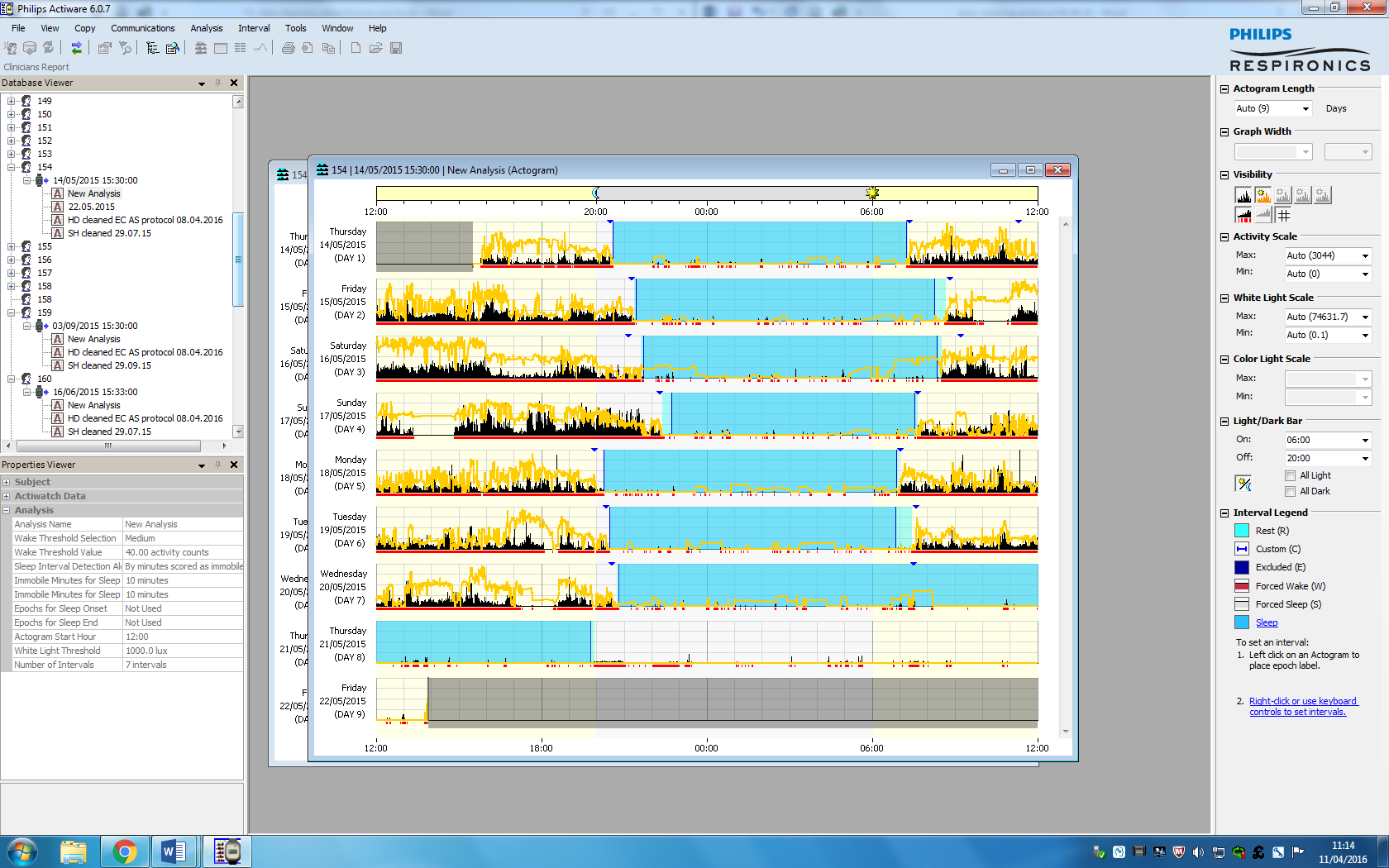


**Parent has reported that child went to sleep at 20:45, woke up at 07:30 but did not get out of bed until 19:45. Total Sleep Time for this night is > twice as long as average Total Sleep Time across the whole week and therefore sleep interval has been excluded.*

**Step-7**: Exclude nights where parents reported that children had a sleepover with a friend

**Step-8:** Change any sleep intervals where it includes parent report sedentary activity.

- Check if the automatically-calculated sleep interval overlaps with the parent reported sedentary activity in the diary.
- If the sedentary activity in the sleep diary ends before the sleep interval in the actigraphy, do not change the sleep interval.
- If sleep interval starts during the period of sedentary activity, delete the sleep interval and create a new sleep interval. Extract the end of the sleep interval from the summary statistics and input the start of the sleep interval using the following guidance:
  - a) If the diary ‘time lights turned off’ and the event marker are congruent (+/- 15 minutes) use the time the event marker was pressed as the start of the newly created sleep interval.
  - b) If the diary ‘time lights turned off’ and event marker are incongruent (> +/- 15 minutes) use the time the event marker was pressed as the start of the newly created sleep interval **unless** the event marker was pressed during the sedentary activity (if this is the case, use the diary ‘time lights turned off’ as the start of the newly created sleep interval).
  - c) If the event marker was **not** pressed, use parent reported ‘time lights turned off’ as the start of the newly created sleep interval.
  - d) If the event marker was not pressed and the parent diary does not report ‘time lights turned off’ use the end of the period of sedentary activity in the sleep diary as the start of the newly created sleep interval.


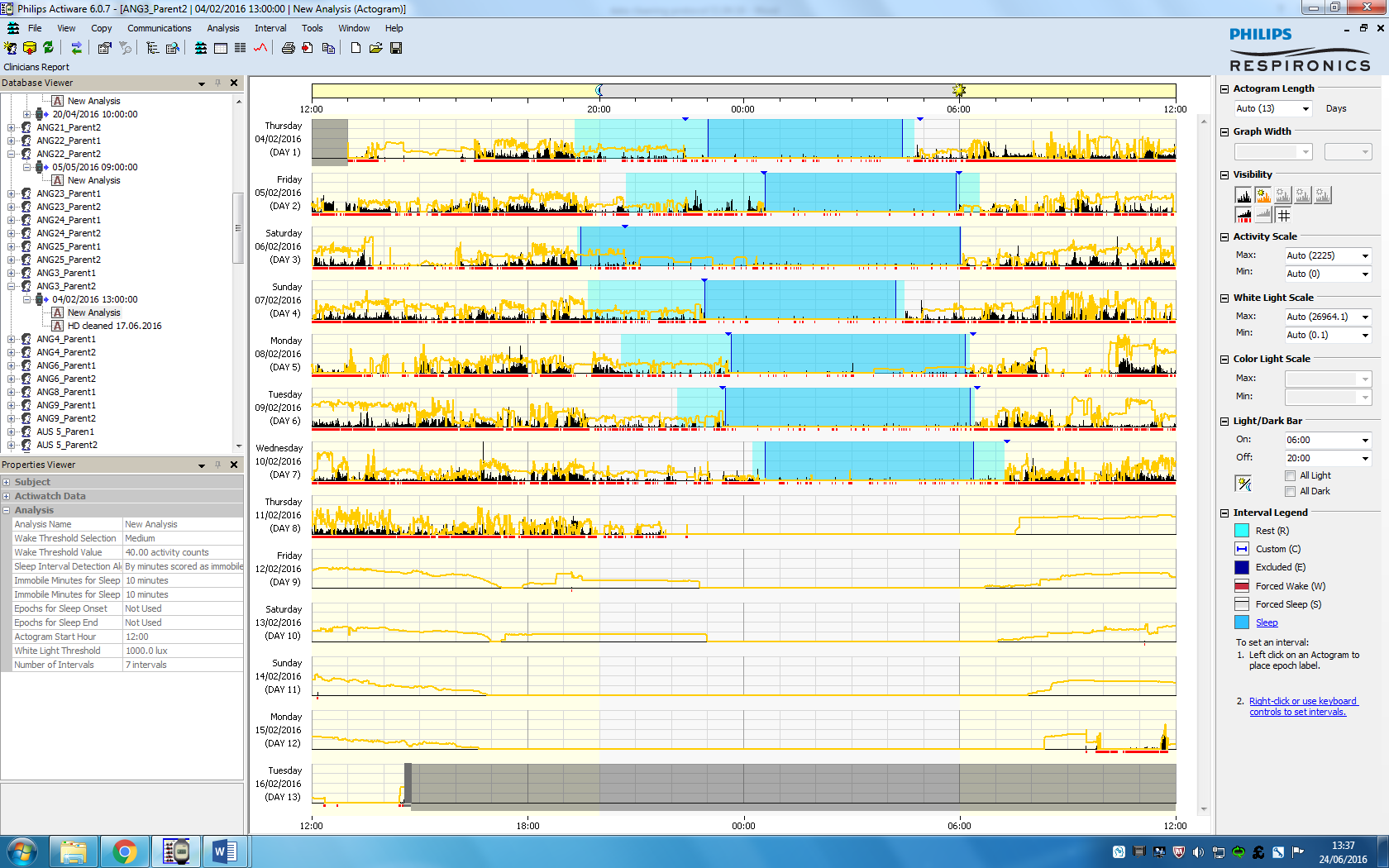


** The automatically-calculated sleep interval suggests that parent has been in bed from 7:30pm on Thursday 4^th^. The parent diary reported sedentary activity between 7:30pm and 11:00pm. The diary and the event marker are incongruent, and the event marker was pressed during the sedentary activity. As a result, we clear this sleep interval and insert a new one using the parent reported ‘time lights turned off’*.


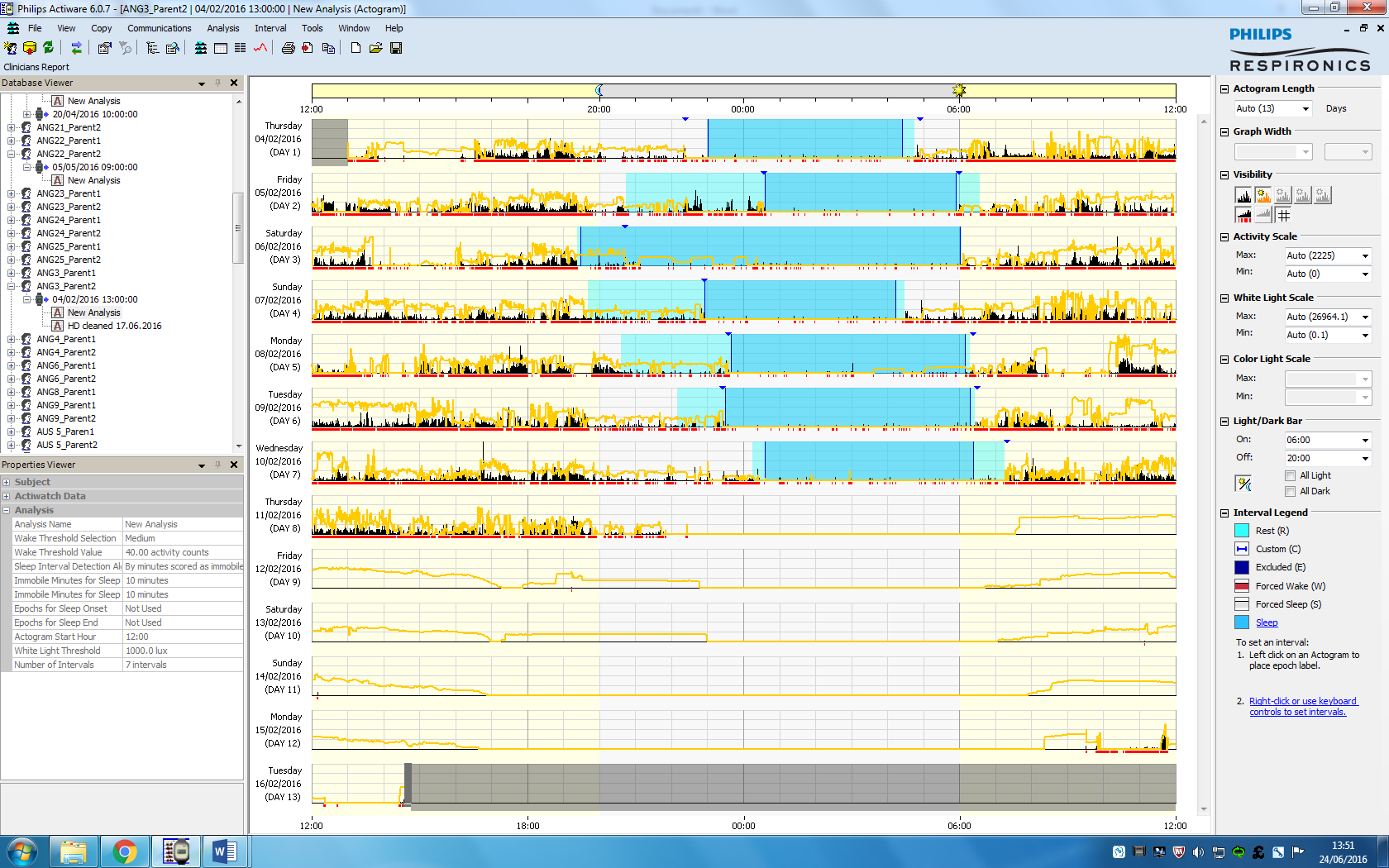


**Step 9 – Changing the start of the rest interval**

- Look over the whole week of the sleep diary. If on the majority of nights (e.g. 4/7) the following apply:
  - Event marker is missing
  - Event marker has been pressed multiple times within two hours of the automatic interval
  - Parent indicated that the event marker was pressed at the incorrect time

Then use the sleep diary to adjust the start of the sleep intervals for each day. Use adjusted algorithms on page 10. For each day that the event marker is inaccurate (even if this is just 1/7) use the adjusted algorithm for that day specifically.

-
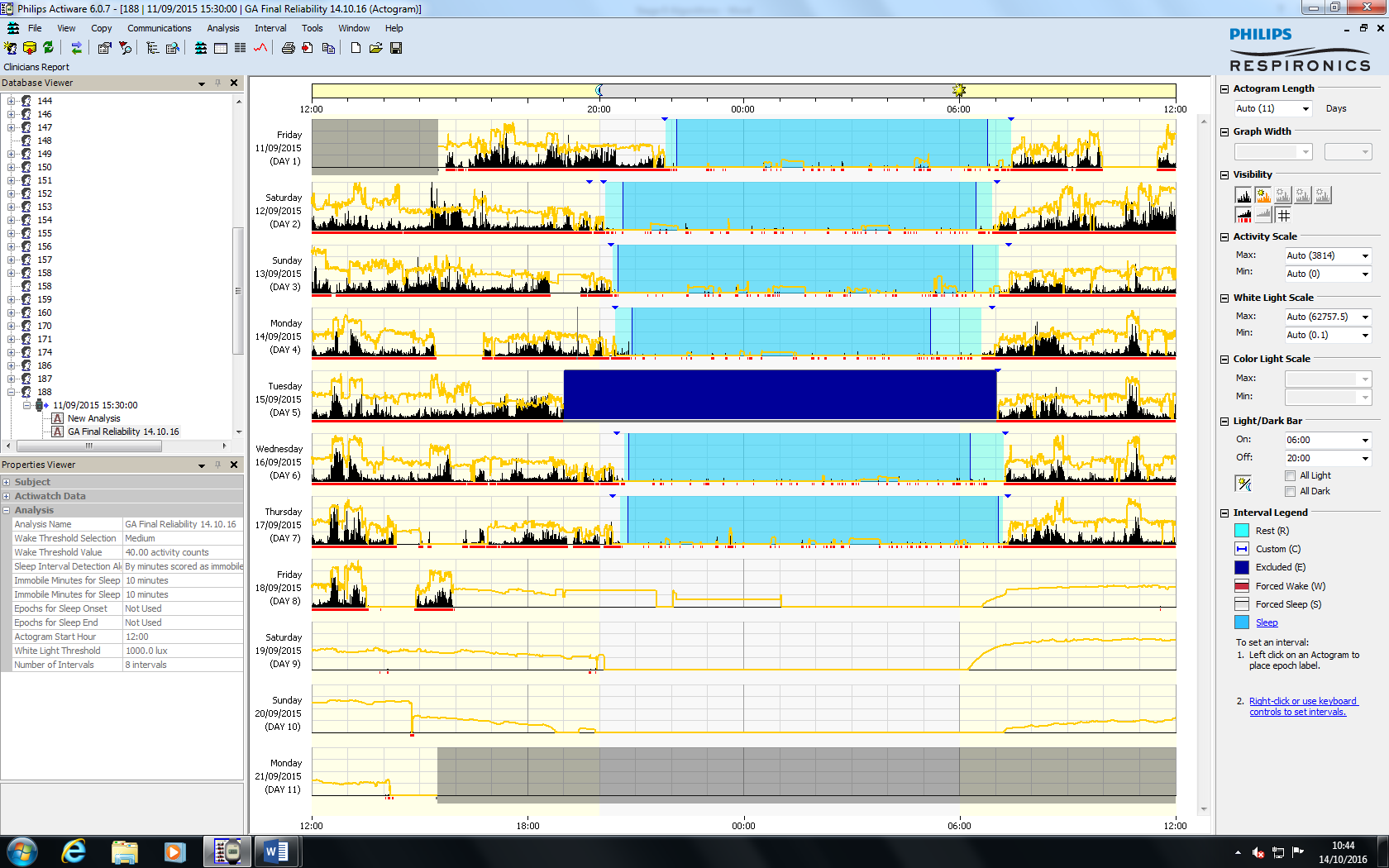
Look at the event marker, sleep diary and the automatically calculated rest interval. If they are concordant (all three within +/-15 minutes of each other) then leave the automatically calculated rest interval.

**The sleep diary, event marker and automatically calculated interval all indicate that the lights were turned off at 21:45, so the automatically calculated interval is left.*

-
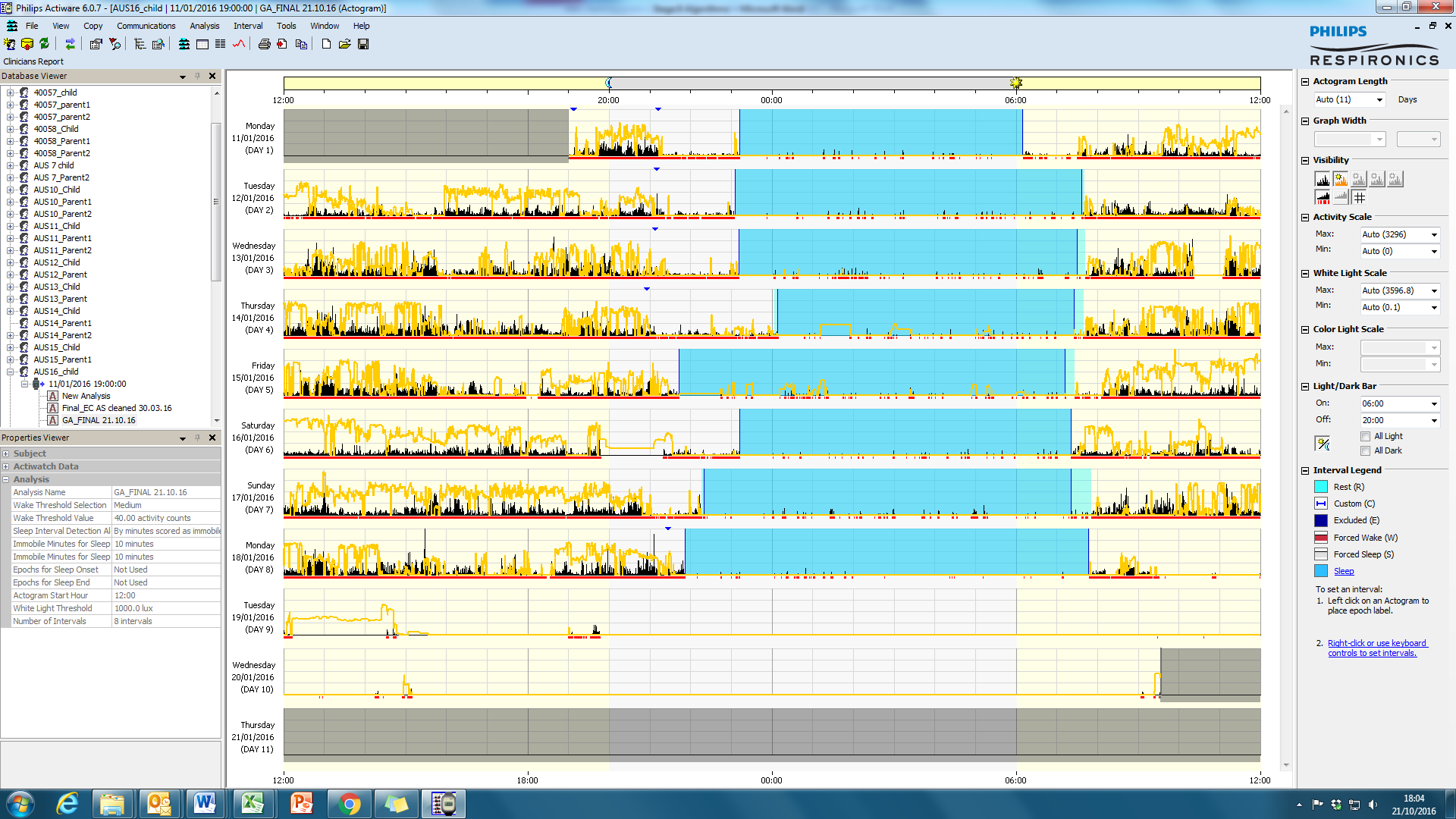
If the event marker and sleep diary are concordant but discordant with the automatically calculated rest interval, delete this interval and replace it with a new interval. The new start time should be the time indicated by the event marker.

*
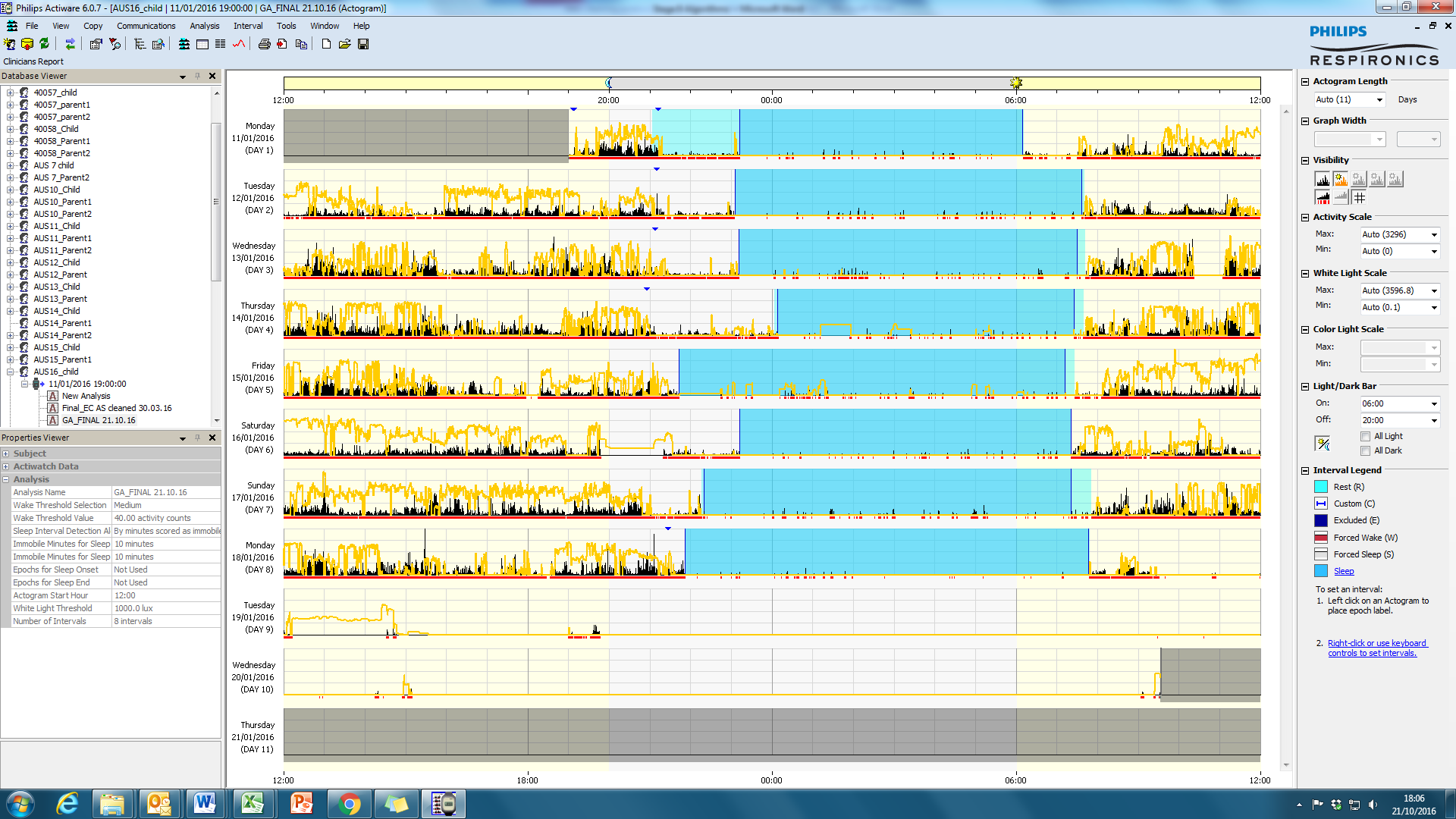
*The parent diary and event marker indicate that the lights were turned off at around 21:15. However, the automatically calculated rest interval begins at 23:13. Since they are discordant, the event marker is used to indicate when the child went to sleep and a new rest interval is added*.

- If the event marker is discordant with the sleep diary but concordant with the automatically calculated rest interval, leave the automatically calculated rest interval.


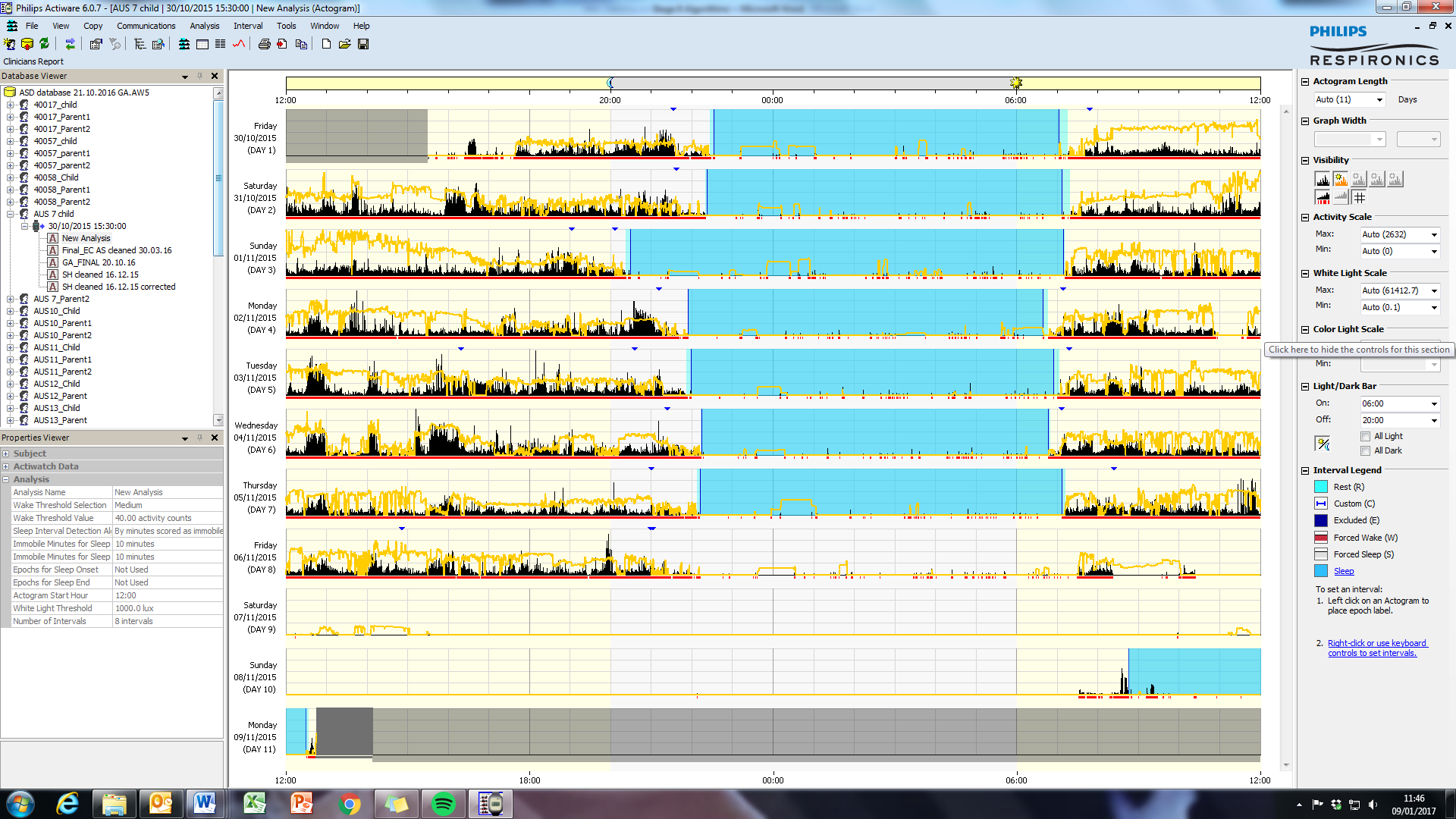


**The event marker has been pressed at 20:06:30, but the parent reports in the sleep diary that they are not sure when it was pressed or when the lights were turned off. The automatically calculated rest interval begins within 15 minutes of the event marker being pressed, so no change is made to the start of the rest interval.*

-
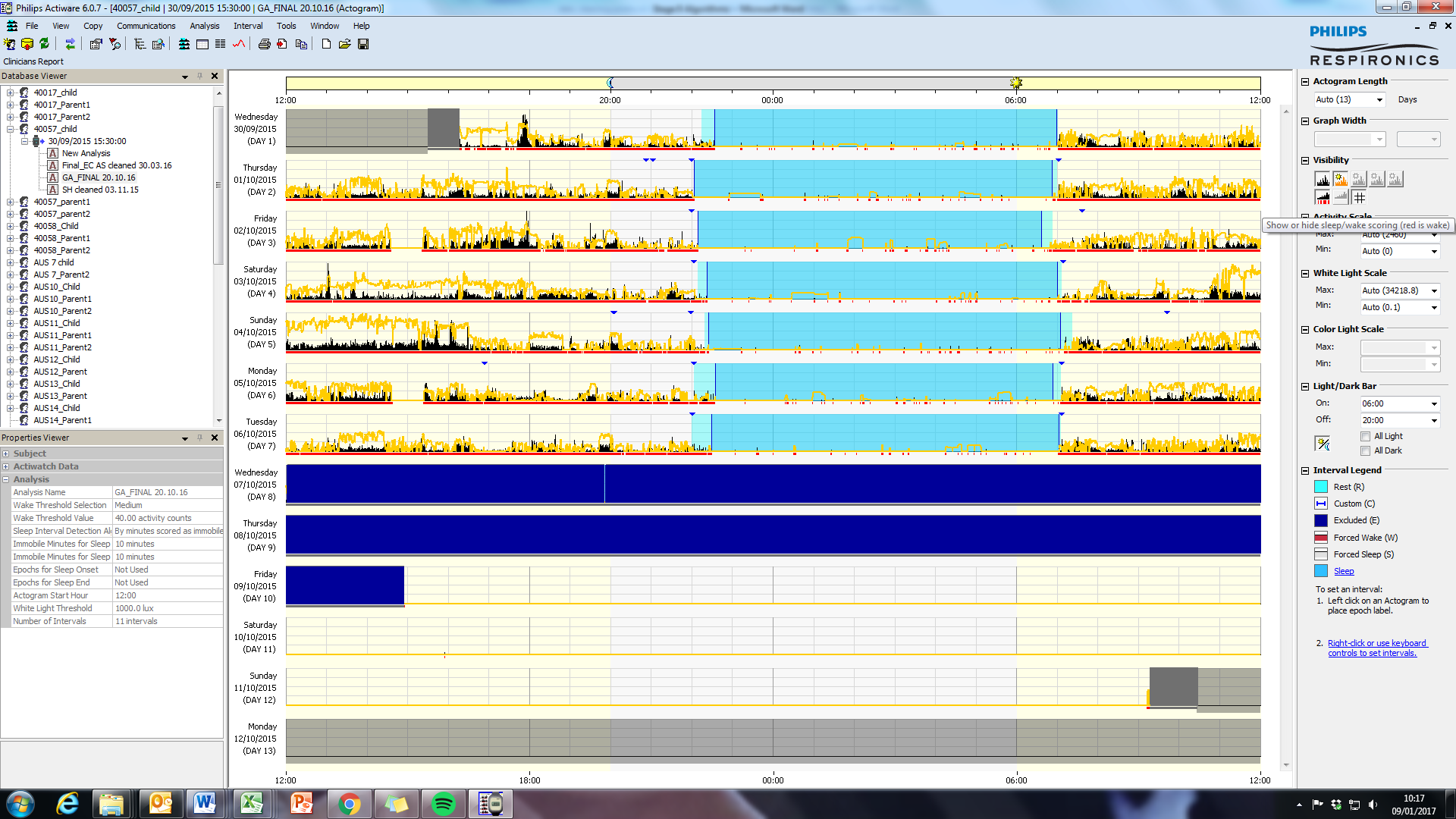
If the sleep diary is discordant with the event marker but concordant with the automatically calculated rest interval, leave the automatically calculated rest interval.

**The sleep diary indicates that the lights were turned off at 22:30, but the event marker has been pressed at 21:59. The automatically calculated interval suggests the child fell asleep at 22:24:30. Since this is within 15 minutes of the time indicated by the parent sleep diary, leave the automatically calculated interval.*

- If the sleep diary, event marker and automatically calculated rest interval are all discordant, leave the automatically calculated rest interval.


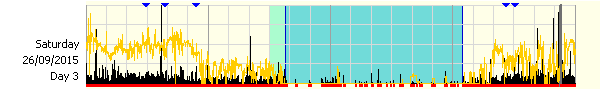


**Sleep diary indicated lights out at 20:15, this is discordant with light and activity levels by 45 mins. Sleep diary lights out time is earlier than automatic interval. No sedentary activity overlap with sleep period- used automatically calculated sleep interval.*


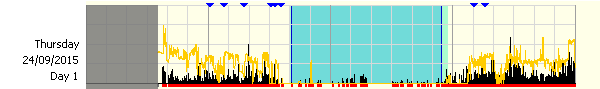


**Sleep diary indicated lights out at 22:30, this is discordant with lights and activity levels by 30 mins. Sleep diary lights out time is later than automatic interval. No sedentary activity overlap with sleep period- used automatically calculated sleep interval*.

ALTERNATIVE ALGORITHMS

- If the event marker has been identified as missing or inaccurate on a majority of nights, then it should be discounted.


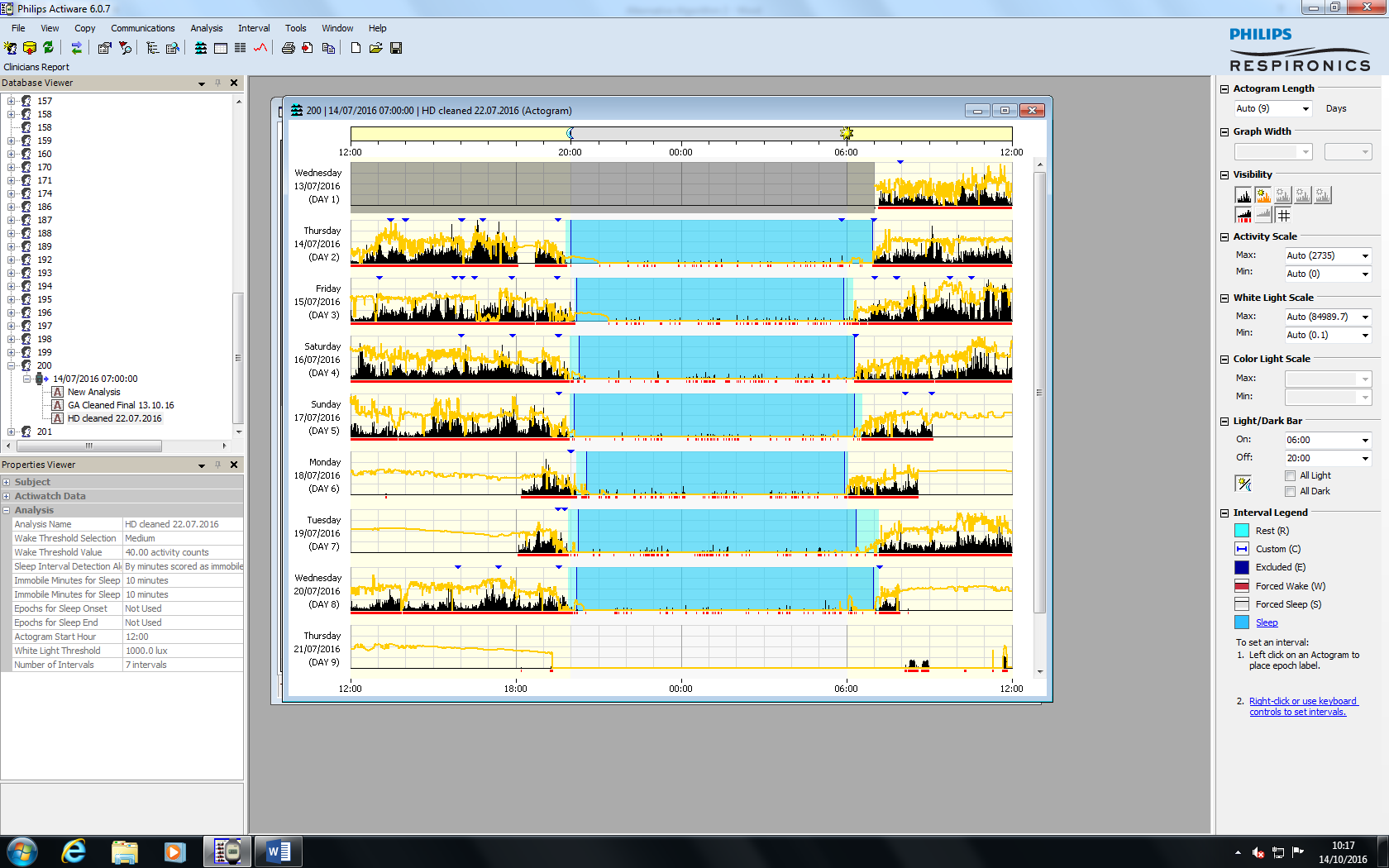


**On several nights (Friday, Saturday, Tuesday) the event marker has been pressed multiple times within two hours of the automatically calculated rest interval. Additionally, on Monday, the event marker has been pressed at 20:02, but the parent diary indicates it was pressed at 19:30. This means that the event marker is inaccurate for the majority of nights (4/7), and so should be discounted. This means that only alternative algorithms 1 and 2 should be used to clean the data.*

-
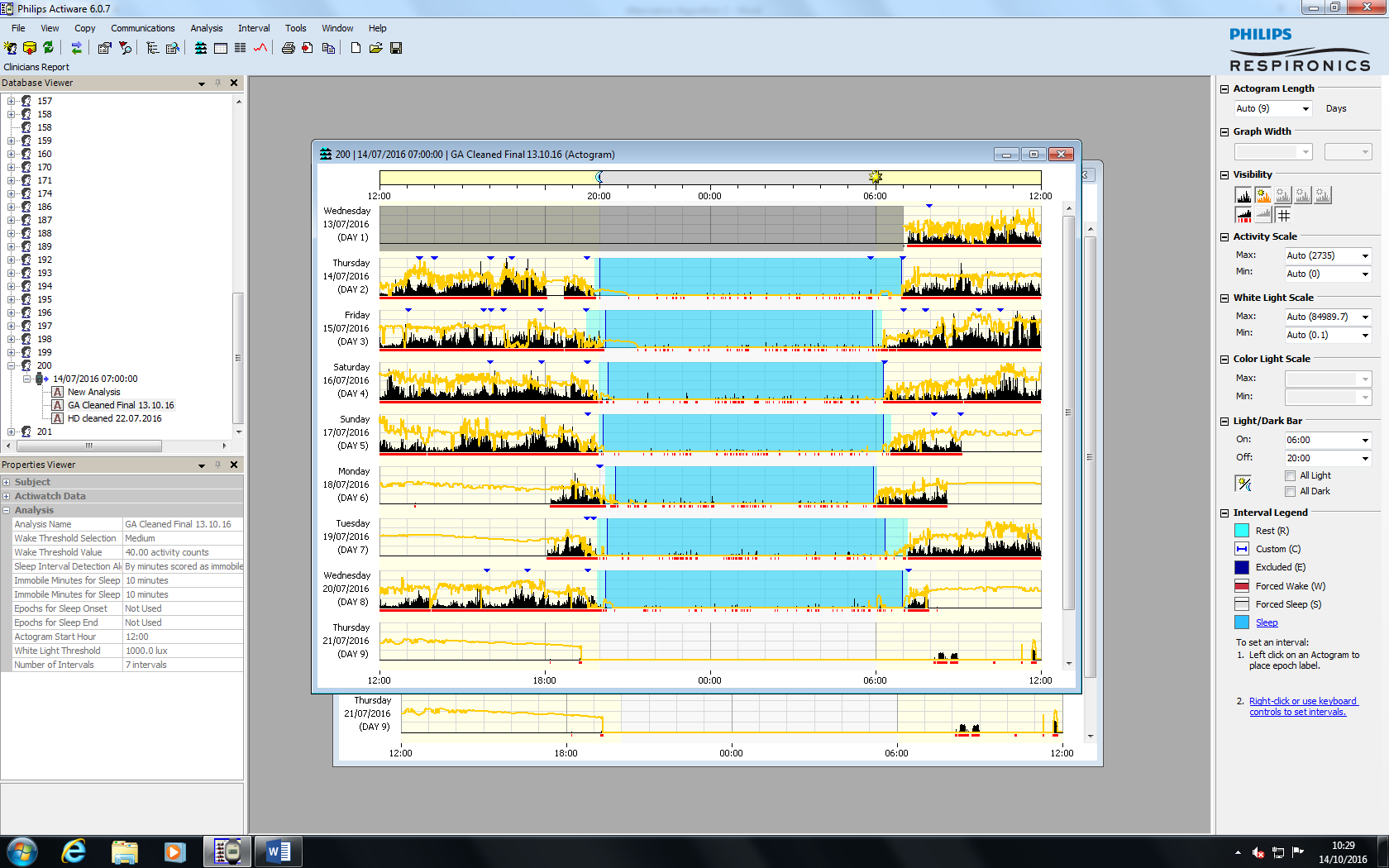
If the event marker has been identified as inaccurate, but the sleep diary and automatically calculated rest interval are concordant, leave the automatically calculated rest interval.

**The event marker has been pressed several times. The sleep diary and automatically calculated interval indicate that the child went to sleep at around 19:45, so the automatically calculated interval is left.*

If the event marker has been identified as inaccurate and the sleep diary is discordant with the *
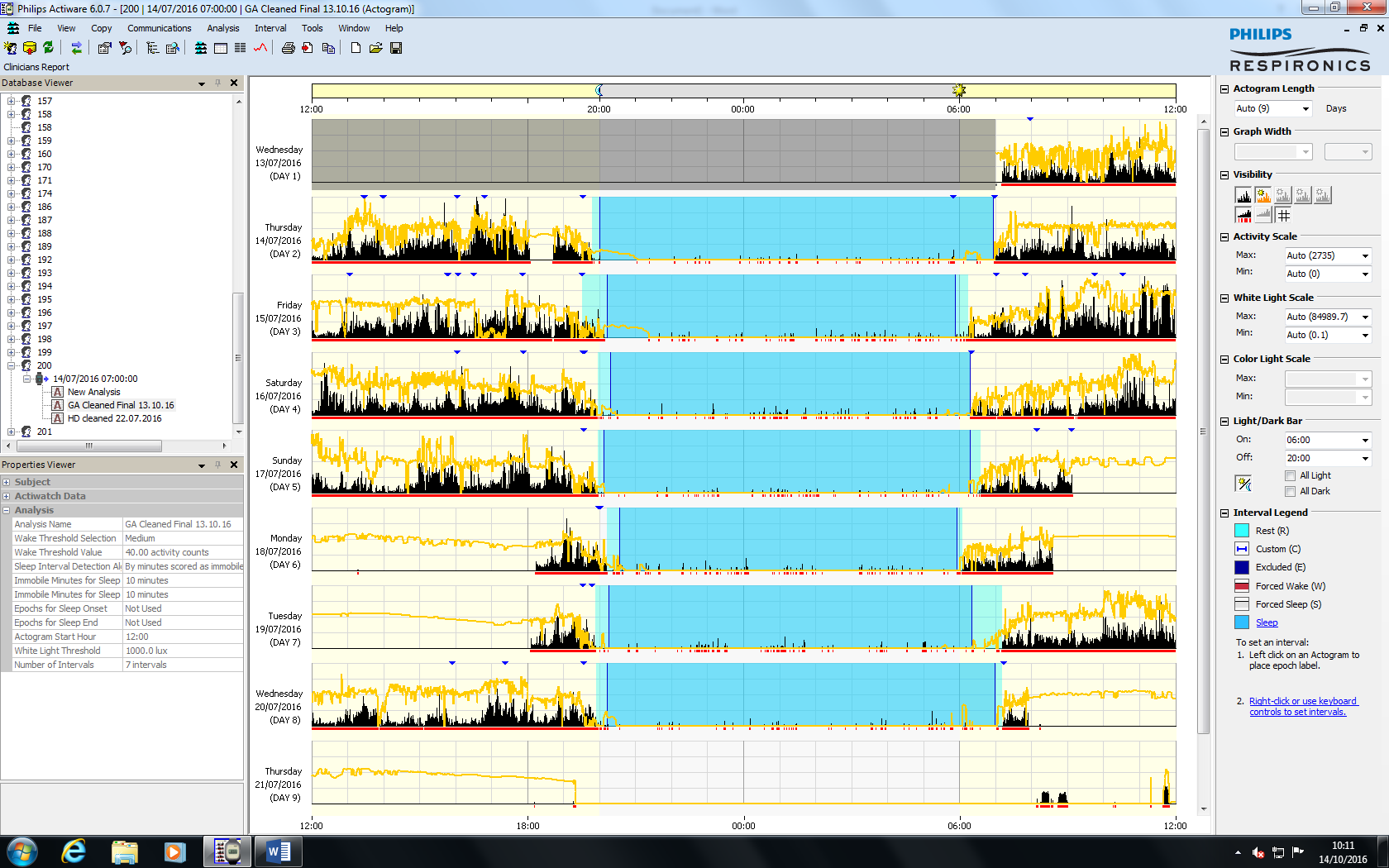
*automatically calculated interval, use the sleep diary to insert a new interval.


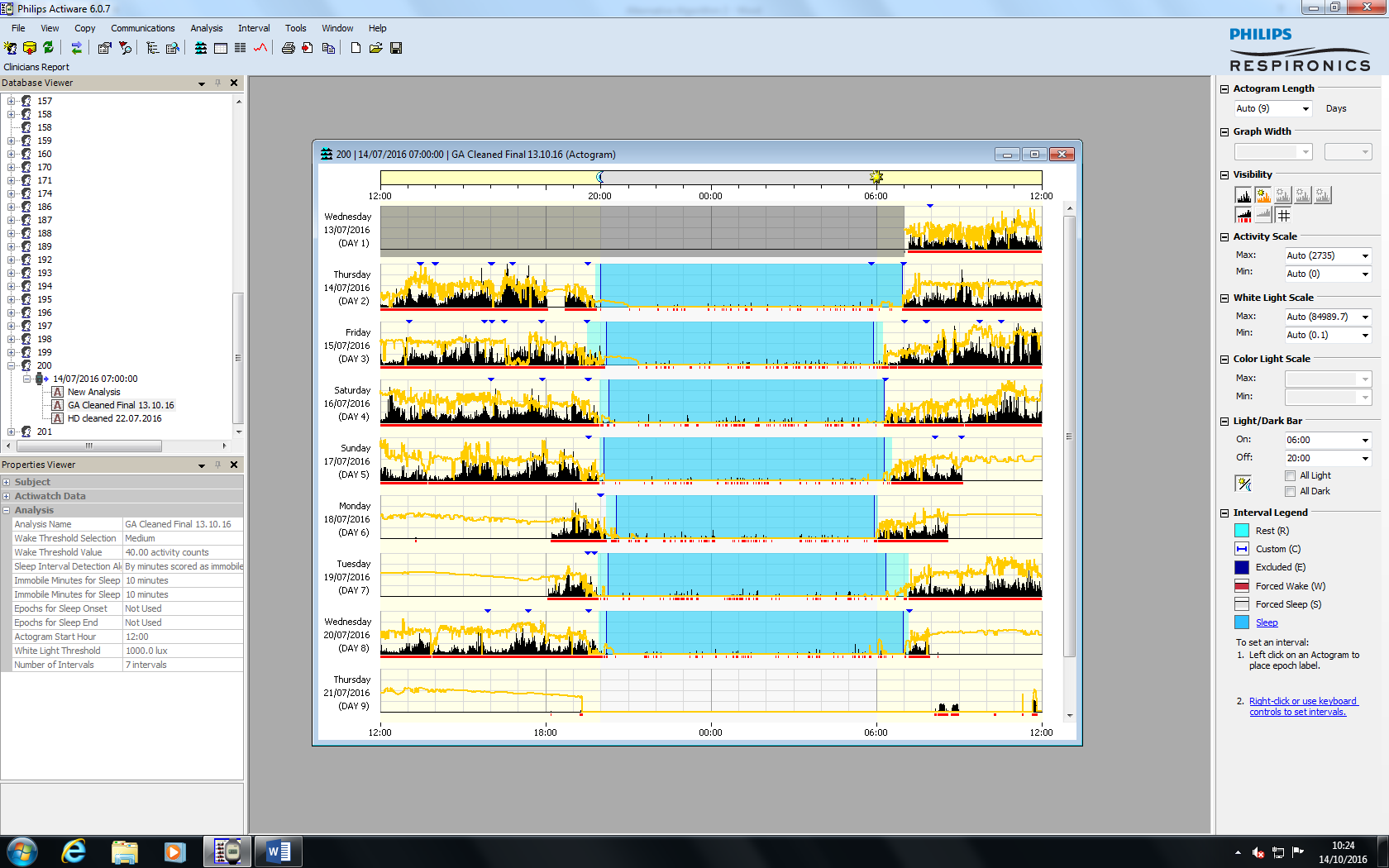
 **The event marker has been pressed multiple times throughout this participant’s sleep week, so the event marker is discounted. The sleep diary says that the lights were turned off at 19:35pm, but this is discordant with the automatically calculated sleep interval by 20 minutes. Therefore, the automatic interval is cleared and a new interval is inserted, which begins at 19:35pm.*

# Supplementary Material 4: Objective Sleep Parameters Derived from Cleaned Actigraphy

| Parameter | Definition |
| --- | --- |
| Bedtime | Clock time child was put to bed with lights out, thus entering a ‘restful’ state. This was determined using the event marker, sleep diary and automatically calculated rest interval to avoid sedentary activity being included in the automatically calculated rest interval. |
| Sleep Onset Latency | Time between bed time and first sleep period (the first ten minutes scored as sleep after Bed Time, according to actigraphy) |
| Get Up Time | Clock time of the end of the final period of sleep in the morning |
| Time in Bed | Time between Bed Time and Get Up Time |
| Total Sleep Time | Total amount of time scored as sleep between sleep onset and Get Up Time |
| Sleep Efficiency | Percentage of Time in Bed spent asleep: Total Sleep Time/ Time in Bed x 100 |
| Wake After Sleep Onset | Number of minutes scored as wake after first period of sleep, according to Actiwatch default settings (medium sensitivity, 40 counts per epoch) |
